# FOXM1 regulates platelet-induced anoikis resistance in pancreatic cancer cells

**DOI:** 10.1186/s12964-025-02644-8

**Published:** 2026-01-14

**Authors:** Alissa Ernesti-Soldatkin, Carolin T. Neu, Beate Heydel, Ferdinand Krannich, Helmut Laumen, Tony Gutschner, Monika Haemmerle

**Affiliations:** 1 Institute of Pathology, Section of Experimental Pathology, Martin Luther University Halle-Wittenberg, Halle, Germany; 2 Department of Internal Medicine I, University Hospital Halle, Martin Luther University Halle-Wittenberg, Halle, Germany; 3 Institute of Molecular Medicine, Section for RNA Biology and Pathogenesis, Martin Luther University Halle-Wittenberg, Halle, Germany; 4 Present Address: Veterinary Faculty, Chair of Biochemistry and Chemistry, Ludwig Maximilian University Munich, Munich, Germany

**Keywords:** Metastasis, Platelet-cancer cell interaction, Anoikis, FOXM1, Pancreatic cancer

## Abstract

**Background:**

Resistance to anoikis, a form of programmed cell death that occurs after detachment from the surrounding extracellular matrix, is a prerequisite for the survival of circulating tumor cells (CTCs) in the bloodstream. Platelets can interact with these CTCs and protect them from cytokine- and immune cell-mediated cell death. Whether platelets can regulate anoikis resistance by controlling intrinsic gene expression changes in tumor cells that contribute to metastasis has not been studied in detail in pancreatic cancer cells.

**Methods:**

Pancreatic cancer cells were cultured under attached or low-attachment conditions to induce and mimic anoikis. The detached cells were co-cultured with platelets and subsequent gene expression analyses were performed to identify deregulated pathways responsible for survival under detached conditions that are mediated by platelets.

**Results:**

We observed a cell line-dependent sensitivity of pancreatic cancer cells to anoikis and that anoikis resistance was greatly enhanced by platelet interaction. RNA sequencing and transcriptome analyses identified FOXM1 as a differentially regulated gene between attached and detached cells, and its expression was modulated by platelets via an activated AKT signaling pathway. Manipulating FOXM1 protein expression via gain- and loss-of-function approaches or by inhibiting its activity using small-molecule inhibitors significantly impacts platelet-influenced death rates. Intriguingly, single-cell RNA sequencing and immunohistochemical analyses revealed higher FOXM1 expression in pancreatic cancer metastases than in primary tumors.

**Conclusion:**

Overall, these findings suggest that targeting FOXM1 may be a promising therapeutic strategy to interfere with the metastatic progression of pancreatic cancer, which might particularly benefit patients with high blood platelet counts.

**Supplementary Information:**

The online version contains supplementary material available at 10.1186/s12964-025-02644-8.

## Background

Pancreatic cancer is a deadly disease with a poor overall survival rate. As of 2022, pancreatic cancer is the sixth leading cause of cancer-related death worldwide, with the highest incidence rates in Europe, North America, and Australia [1]. A main reason for the high mortality rate is late diagnosis, and most patients already have local or distant metastases [2]. Metastasis is a highly complex process involving detachment of cancer cells from the primary tumor, intravasation into the blood and lymphatic vessels, extravasation into distant organs, and ultimately, the ability to form a secondary mass [3]. For the successful formation of distant metastases, the survival of circulating tumor cells (CTCs) in the blood is crucial. However, the majority of CTCs die due to anoikis, an apoptotic cell death program induced by disruption of epithelial cell-matrix interactions [4]. During their short transit time, CTCs encounter several challenges that affect their survival, including shear stress and contact with immune cells. It has been suggested that the survival of CTCs can be greatly enhanced by their interaction with platelets that form a platelet coat on the CTC surface, thereby physically protecting cancer cells from immune and physical stressors [5]. These microclusters of platelets and tumor cells have high metastatic potential, as various studies have shown that platelets can promote metastasis in cancer [6, 7]. However, whether platelets enhance the survival of pancreatic cancer cells under low-attachment conditions, thereby contributing to metastasis, has not been studied in detail. Here, we sought to determine the underlying mechanisms relevant for the survival of pancreatic cancer cells after detachment and evaluated the effects of platelets as mediators of anoikis resistance. Our functional analyses revealed that the AKT-FOXM1 axis is crucial for the survival of detached cells. In particular, we found that FOXM1 modulates the transcriptional response, thereby contributing to platelet-induced anoikis resistance in pancreatic cancer cells in vitro. Moreover, our transcriptional and immunohistochemical analyses revealed increased FOXM1 expression in metastases compared with primary tumors and that FOXM1 levels were associated with survival in patients with pancreatic ductal adenocarcinoma (PDAC). Hence, these findings highlight FOXM1 as a promising target for treating metastatic disease, particularly in patients with high platelet counts.

## Materials and methods

### Cell culture

CAPAN-1 (ACC 244), MIA Paca-2 (ACC 733), PANC-1 (ACC 783), PA-TU-8988 S (ACC 204) and PA-TU-8988T (ACC 162) pancreatic cancer cells were obtained from the DSMZ. BxPC-3 (CRL-1687) and SU.86.86 (CRL-1837) pancreatic cancer cells were obtained from ATCC. IMIM-PC1 cells (CVCL_4061 [8]), were a gift from Prof. Michl (Internal Medicine I, University Hospital Halle). The cell lines were routinely tested for mycoplasma contamination via Mycoplasma-specific PCR. The human pancreatic cancer cell lines BxPC-3 and SU.86.86 were cultured in RPMI-1640 supplemented with 10% fetal bovine serum (FBS) and 1% penicillin-streptomycin. The human pancreatic cancer cell lines CAPAN-1, IMIM-PC1, MIA PaCa-2, PANC-1, PA-TU-8988S, and PA-TU-8988T were cultured in DMEM supplemented with 10% fetal bovine serum (FBS) and 1% penicillin-streptomycin. The cells were maintained at 37 °C in a humidified incubator infused with 20% O_2_ and 5% CO_2_.

### SiRNA transfection

For experiments involving small interfering RNA (siRNA) transfection, control siRNA and two independent FOXM1 siRNAs were transfected (40 nM final concentration) using RNAiMAX (Thermo Fisher Scientific). siRNA transfections were performed 24 h before the cells were seeded into low-attachment plates and co-cultured with platelets. The cells were collected 48 h later for RNA expression analysis and 72 h later for propidium iodide (PI) staining and flow cytometry analysis. The following siRNAs were used in this study: AllStars Neg. Control siRNA (Qiagen), FOXM1 siRNA 1: AUAUUCACAGCAUCAUCAC, FOXM1 siRNA 2: GGACCACUUUCCCUACUUU.

### Generation of FOXM1 overexpressing BxPC-3 cells

The FOXM1 overexpression plasmid was generated by cloning the FOXM1 coding sequence into a modified pHAGE vector (modified pHAGE-SMAD4 vector; #116791 from Addgene; kind gift from Gordon Mills & Kenneth Scott [9]. The SMAD4 insert was replaced by a multiple cloning site containing BsrGI, NcoI, NheI, BmtI, NotI, PspXI, XbaI, BstBI and MluI. The FOXM1 coding sequence, including a Flag-tag, was obtained from a Flag-FOXM1 plasmid (#153136 from Addgene; kind gift from Stefan Koch [10]) and removed via the NcoI and XbaI restriction enzymes. Lentiviral particles were produced from HEK293T cells, cultured in DMEM, and transfected with packaging and VSV-G envelope-expressing plasmids (#12259 and #12260 from Addgene; kind gift from Didier Trono). HEK293T supernatants were harvested 48 h after transfection, and BxPC-3 cells were transduced immediately with fresh virus particles. Positive selection with 1 µg/mL puromycin (cat. no. A1113803, Thermo Fisher Scientific) started 24 h after transduction. Overexpression was analyzed at the RNA and protein levels using RT-qPCR and western blotting.

### Cell cycle analysis

BxPC-3 cells were transfected with siRNAs as described above. The cells were harvested 48 h after transfection and fixed in 70% ethanol at 4 °C. The cells were pelleted via centrifugation, washed three times with PBS supplemented with 1% FBS and 1 mM EDTA, and incubated with 100 µg/ml RNase A (cat. no. 12091021, Thermo Fisher Scientific) and 50 µg/ml PI at room temperature for 10 min in the dark. The DNA content of 20,000 cells per sample was measured via flow cytometry using a MACSQuant^®^ Analyzer 10 (Miltenyi Biotec). The results were analyzed using the FlowJo™ software (version 10.8.1).

### Isolation of murine and human platelets

Platelets were isolated as previously described [6]. Briefly, whole blood was drawn from the inferior vena cava of anesthetized athymic nude mice into a 1 ml syringe that was preloaded with 100 µl of acid citrate dextrose solution B (GT Biosciences). Afterwards, the blood was gently mixed with 500 µl of Tyrode’s buffer. Blood was centrifuged at 120 × g for 6 min at room temperature. The platelet-rich plasma fraction was passed through a filtration column of Sepharose CL-2B beads (GE Healthcare) loaded into a siliconized glass column with a 10 μm nylon net filter (Millipore). The cloudy eluents containing platelets were collected in buffer I in a 15 ml Falcon tube. For the isolation of human platelets, blood was drawn into a collection tube containing citrate buffer. Immediately afterwards, the blood was transferred to a 15 ml tube, and equal amounts of Tyrode’s buffer was added. The sample was subsequently centrifuged at 200 × g for 20 min at room temperature. The platelet-rich plasma fraction was transferred to a new tube containing equal amounts of buffer I. To remove contaminating red and white blood cells, the plasma was centrifuged again at 100 × g for 20 min at room temperature. The supernatant containing the platelets was transferred into a new tube, and the platelets were pelleted by centrifugation at 800 × g for 15 min. For both, murine and human platelets, the number of platelets was counted with a hemocytometer and the platelets were immediately used for subsequent experiments. For the indicated experiments, the platelets were treated with 1 U/ml of thrombin (cat. no. T6884, Sigma-Aldrich) and 5 mM CaCl_2_ to ensure complete activation and platelet degranulation. Clotted platelets were spun down at 1,500 × g for 10 min, and the supernatant containing the platelet releasate was added to the cancer cells.

### In vitro low-attachment/anoikis assay

On the first day, 500,000 cells were seeded in an ultra-low attachment 6-well plate (cat. no. 3471, Corning). Immediately thereafter, the platelets were isolated as described above and 100 × 10^6^ platelets diluted in buffer I were added to the cancer cells. For all platelet co-culture experiments, an equal amount of buffer I was added to the control cells. Two or 48 h after platelet addition, the cells were lysed for RNA or protein analysis. The number of PI-positive cells 72 h after platelet addition was evaluated via a MACSQuant^®^ Analyzer 10 (Miltenyi Biotec). In indicated experiments, 5 µM of the FOXM1 inhibitor FDI-6 (cat. no. S9689, Selleck Chemicals), 10µM of Thiostrepton (cat. no. S4354, Selleck Chemicals) or 1 µM of the AKT inhibitor MK-2206 (cat. no. S1078, Selleck Chemicals) was added at the time of cell seeding.

### RNA isolation, cDNA synthesis, and quantitative real-time PCR analysis

The cells were harvested using 1 mL TRIZOL as previously described [11]. The isolated RNA was resuspended in 20–30 µL of ultrapure water. For cDNA synthesis, up to 1 µg of total RNA was reverse transcribed via random hexamer primers and M-MLV reverse transcriptase (Promega). Gene expression was measured using the primaQUANT CYBR qPCR mastermix (Steinbrenner Laborsysteme GmbH) with a Light Cycler^®^ 480 II (Roche). The sequence information for the RT-qPCR primers is listed in Table 1.

**Table 1:** List of RT-qPCR primers used in this study

Gene	primer direction	primer sequence (5’ > 3’)
RPLP0	forward	GGCGACCTGGAAGTCCAACT
RPLP0	reverse	CCATCAGCACCACAGCCTTC
FOXM1	forward	TCACAGCAGAAACGACCGAA
FOXM1	reverse	TCACCGGGAACTGGATAGGT
KIF20A	forward	GTTGAAACTCCAAGGCCAGG
KIF20A	reverse	GCTGCAGTCTGTTGAGCTTT
KIF2C	forward	TGTCTCAGAGCTTCGCATCA
KIF2C	reverse	TCGGATGGAATGGACTTGCT
KIF4A	forward	CTGCAGCCCATTCAGTACCA
KIF4A	reverse	CGCTCACTCAACTTGGCTTG
CCNA2	forward	GGACCTTCACCAGACCTACC
CCNA2	reverse	GTGTCTCTGGTGGGTTGAGG
CCNB2	forward	CGACGGTGTCCAGTGATTTG
CCNB2	reverse	TGGTGGGTTGAACTGGAACT
CDC20	forward	GAGGTGCAGCTATGGGATGT
CDC20	reverse	ACATCATGGTGGTGGATGTG
BIRC5	forward	CATGGCTACCAGCACCTGAA
BIRC5	reverse	AGCCCAGAAGCCTCATTCAC
AURKB	forward	GGAGTGCTTTGCTATGAGCTGC
AURKB	reverse	GAGCAGTTTGGAGATGAGGTCC

### RNA sequencing

RNA was isolated from BxPC-3 and SU.86.86 cells under low-attachment or attached conditions with or without platelet co-incubation for 48 h as described above. mRNA-sequencing was performed with 2 µg of total RNA (*n* = 3) for each sample. Library preparation and sequencing were performed by Genewiz (Leipzig, Germany). Library preparation was based on polyA selection, and sequencing was performed on an Illumina NovaSeq platform, resulting in approximately 20million reads per sample. Trim Galore! v0.4.3.1 was used for the quality check (80% bases Q ≥ 30). Mapping to the human genome hg38 followed by differential gene expression analysis was performed using RNA STAR v2.6.0b-2 and edgeR v 3.24.1.

### Gene set enrichment analysis and protein-protein interaction prediction

Differentially expressed genes in BxPC-3 and SU.86.86 cells under attached versus low-attachment conditions were ranked according to their fold change (FC) and gene overlap between both cell lines was generated after a FC cut-off of |log2(FC)| > 2, FPKM (fragments per kilobase of transcripts per million mapped reads) cut-off of 2 (in at least three samples) and FDR (False Discovery Rate) cut-off ≤ 0.05. Overlap with genes regulated under low-attachment conditions with or without platelet co-incubation was performed using an FC cut-off of |log2(FC)| > 1 and a FPKM cut-off of 2 (in at least three samples). For GSEA, the differentially expressed genes were pre-ranked according to their log2(FC) values and analyzed using the GSEA software [12, 13]. Results with an FDR q-value of ≤ 0.05 were considered statistically significant. Furthermore, prediction of protein-protein interactions was performed using STRING (https://string-db.org) with a minimum required interaction score of 0.7 [14].

### Transcriptome/Survival analysis using patient data

For transcriptome analysis of patient data as well as survival analysis, published and online available data were used [15, 16]. These datasets were accessed and downloaded via R2: Genomics Analysis and Visualization Platform (https://r2.amc.nl).

### Single cell RNA sequencing analysis

Raw count matrices were obtained from the Gene Expression Omnibus (GEO) GSE205013 [17]. Count matrices were merged and pre-processed using Seurat (v5.0.3) [18], including quality control (≥ 200 nFeature_RNA, ≥ 1000 nCount_RNA, < 20%.mt), doublet detection (scDblFinder v1.16.0) [19], cell cycle scoring, normalization (SCTransform, v2 regularization) [20], and principal component analysis (PCA). Cells were clustered using Seurat’s implementation of the Leiden algorithm, annotated on the basis of canonical marker expression and visualized using uniform manifold approximation and projection (UMAP). Copy number variations (CNVs) were inferred using inferCNV (v1.18.1) [21], employing T/NK cells and fibroblasts as a reference. Cells were then scored based on the relative sum of inferred CNVs (0.95 > expr.data > 1.05), with epithelial cells above the 95th percentile of CNVs inferred in the reference being classified as malignant. *FOXM1* expression in primary tumors and metastases was tested using a Wilcoxon rank-sum test, with a FDR-adjusted p-value ≤ 0.05 being considered to indicate significance.

### Protein analysis via Western blotting and protein array

Cells were harvested by lysing the cell pellet with RIPA buffer (50 mM Tris-HCl pH 8.0, 150 mM NaCl, 1% (v/v) IGEPAL CA-630, 0.5% (w/v) Na-deoxycholate, 0.1% (w/v) SDS), supplemented with 10% protease and phosphatase inhibitors (cat. no. A32957, Thermo Fisher Scientific, cat. no.11836153001, Roche). SDS-polyacrylamid gel electrophoresis (SDS-PAGE) was performed at 120 V and proteins were transferred onto a nitrocellulose membrane using the wet blot method. Detection of protein expression was performed using Bio-Rad ChemiDoc MP (Bio-Rad Laboratories). Protein expression was quantified using ImageJ [22]. The following primary antibodies were used: FOXM1 (clone D12D5; #5436), cleaved PARP (clone D64E10; #5625), phospho-AKT (Ser473, clone D9E; #4060), AKT (clone C67E7; #4691), all from Cell Signaling Technology; GAPDH (clone GAPDH-71.1, #G8795, Sigma-Aldrich); RPL7 (#A300–741 A, Thermo Fisher Scientific). The secondary antibodies (IRDye^®^ 800CW Donkey anti-Rabbit; IRDye^®^ 680RD Donkey anti-Mouse) were fluorescently labelled and purchased from LI-COR Biosciences GmbH. To evaluate the phosphorylation status of 49 receptor tyrosine kinases in BxPC-3 cells, the Proteome Profiler Human Phospho-RTK array kit (cat. no. ARY001B, Bio-Techne GmbH) was used according to the manufacturer’s instructions.

### Immunofluorescence

Suspension cells were spun onto glass microscope slides pretreated with poly-L-lysine (cat. no. P8920, Sigma-Aldrich) at 5000 rpm for 5 min. The slides were dried for another five minutes. Thereafter, the cells were fixed in 4% paraformaldehyde for 30 min, followed by washing in phosphate-buffered saline (PBS) and incubation with blocking buffer (1×PBS/1% BSA/0.3% Triton™ X-100) for 60 min. Incubation with a FOXM1 primary antibody (clone D12D5; #5436; Cell Signaling Technology), diluted 1:100 in blocking buffer, was performed overnight at 4 °C. The next day, the slides were washed with PBS and incubated for 1 h with a secondary α-rabbit AlexaFluor^®^ 488 antibody (Jackson ImmunoResearch Laboratories), diluted 1:500 in blocking buffer. The actin cytoskeleton was visualized via phalloidin staining (P1951; Sigma-Aldrich Chemie GmbH), and the nuclei were stained with DAPI (1 µg/mL; Carl Roth). After a final wash, the slides were mounted with Mowiol (Calbiochem). Pictures were taken using a Keyence BZ-X microscope.

### Immunohistochemistry

Immunohistochemical staining for FOXM1 was performed on formalin-fixed, paraffin-embedded tissue sections from primary PDAC and corresponding peritoneal metastases. Three- to five-micron-thick sections were deparaffinized, rehydrated, and subjected to head-induced antigen retrieval in Tris-EDTA buffer (pH 9.0). Endogenous peroxidase activity was blocked with 3% hydrogen peroxide, followed by a blocking step and incubation with a rabbit monoclonal anti-FOXM1 antibody (1:500; clone D3F2B; #20459, Cell Signaling Technology) at 4 °C overnight. Detection was achieved via a polymer-based and HRP-conjugated secondary system (cat. no. POLHRP-100, Zytomed Systems) and visualized using DAB as a chromogen. The slides were counterstained with hematoxylin. For each case, five high-power fields (HPFs, 200x magnification) were randomly selected within viable tumor areas, avoiding necrotic regions. For each HPF, the percentage of tumor cells showing positive nuclear staining was evaluated, along with the staining intensity (0: no staining; 1+: weak staining; 2+: moderate staining; 3+: strong staining). The result for each sample was expressed using the histoscore (H-score). Scale bar = 200 μm.

### Statistical analysis

All experiments were repeated at least three times. Statistical analysis was performed using Excel and GraphPad Prism 8. Differences between groups were evaluated using two-tailed Student’s t-test, the Mann-Whitney U test or one-way analysis of variance (ANOVA), adjusting for multiple comparisons. The results are presented as the means ± SEM. For all the statistical analyses, *p* ≤ 0.05 was considered statistically significant.

## Results

### Platelets induce anoikis resistance in pancreatic cancer cells

**Fig. 1:**
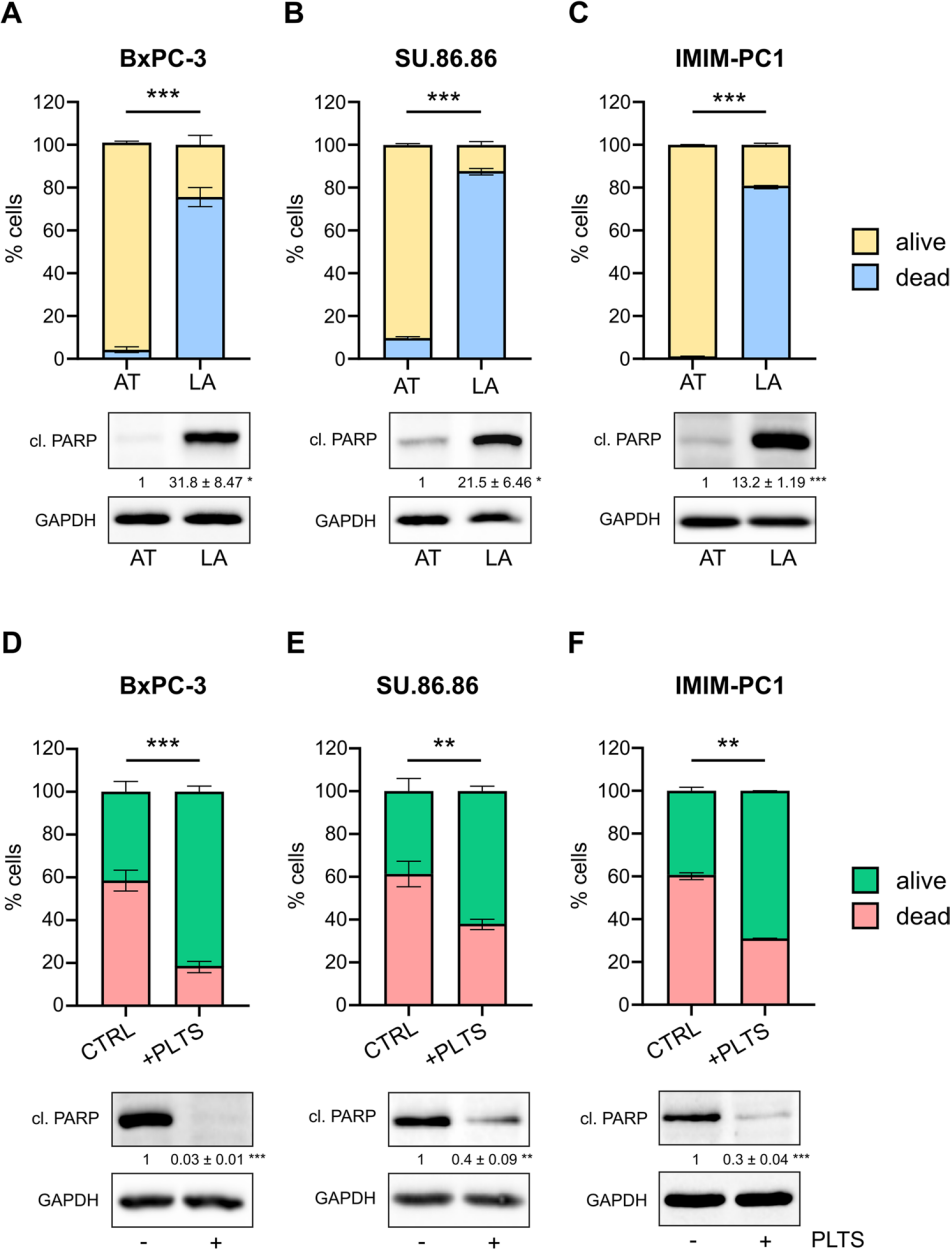
Anoikis rates change under low-attachment conditions and platelet co-incubation. Pancreatic cancer cells were cultured under attached (AT) and low-attachment (LA) conditions for 72 h, and the percentages (%) of dead (= PI-positive) and living (= PI-negative) BxPC-3 (**A**), SU.86.86 (**B**), and IMIM-PC1 (**C**) cells were measured using flow cytometry (upper bar graphs). In addition, increased apoptosis rates were confirmed using protein analysis and quantification of cleaved PARP (lower blots). GAPDH was used as a loading control. **D-F** The corresponding cell lines were co-incubated with 100 × 10^6^ platelets (PLTS) and flow cytometry analysis and western blotting was performed as described above. Bars and error bars represent the mean values and the corresponding SEMs. For western blot analyses, the mean intensity values and the corresponding SEMs relative to those of the controls are shown (*n* = 3–4; *p ≤ 0.05, **p ≤ 0.01, ***p ≤ 0.001).

The survival of cancer cells in the bloodstream and resistance to cell death occurring after detachment from the extracellular matrix are essential for subsequent metastasis. To model non-adherent cultures and examine anoikis rates, we incubated human pancreatic cancer cell lines in ultra-low attachment cell culture plates. Cell death occurring 72 h after cell seeding was used as the major endpoint for measuring anoikis levels in vitro, as previously described [6]. We measured a significant number of dead (PI-positive) cells after detachment in all analyzed cell lines (Fig. 1A-C, Supplementary Fig. 1 A, upper bar graphs). Interestingly, individual differences in anoikis rates between cell lines were detected. While PA-TU-8988T cells (Supplementary Fig. 1E) were highly resistant to anoikis with only 15–20% PI-positive cells after 72 h, BxPC-3, SU.86.86, and IMIM-PC1 cells (Fig. 1A-C) were very sensitive to the low-attachment culture conditions, with apoptosis rates ranging from 75% to 90%. In addition to the flow cytometry results, increased apoptosis rates were confirmed by western blot analyses of the respective cell lines under attached and low-attachment conditions. Specifically, high levels of cleaved PARP were detected in BxPC-3, SU.86.86 and IMIM-PC1 cells (Fig. 1A-C, lower blots), whereas only minor differences in low-attachment cultures compared with attachment cultures were observed in PA-TU-8988T cells (Supplementary Fig. 1E, lower blots). As platelets have been shown to facilitate metastasis in vivo [6, 7], we examined whether platelets could improve the survival of pancreatic cancer cells under low-attachment conditions. Therefore, we co-cultured cancer cells that were initially sensitive to anoikis with platelets and measured the percentage of PI-positive cells after 72 h. Co-incubation of BxPC-3, SU.86.86, and IMIM-PC1 cells with platelets significantly decreased the number of dead detached cancer cells in all tested cell lines and increased survival rates up to 60% (Fig. 1D-F, upper bar graphs), and cleaved PARP levels markedly decreased after tumor cell-platelet co-incubation (Fig. 1D-F, lower blots). To determine whether platelets have similar effects on the highly anoikis-resistant cell line PA-TU-8988T, we repeated the experiments with this cell line and found that anoikis rates and cleaved PARP levels did not significantly change upon platelet co-culture (Supplementary Fig. 1 F).

### Gene expression signatures upregulated in attached cells and by platelets

**Fig. 2:**
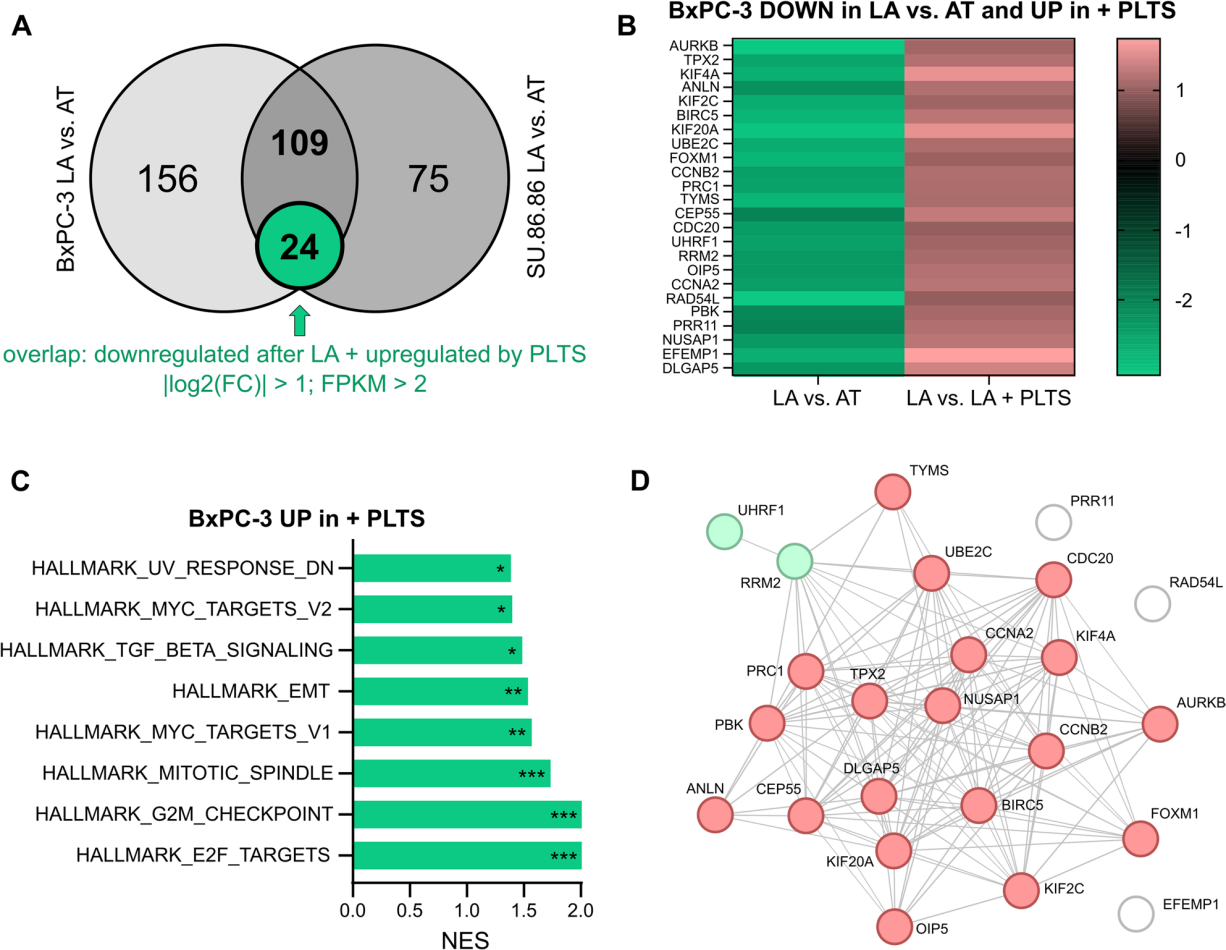
Platelets induce a FOXM1-specific gene signature in cancer cells. **A** RNA sequencing analysis of cells under low-attachment (LA) versus attached (AT) conditions revealed 265 and 184 differentially regulated genes in BxPC-3 and SU.86.86 cells, respectively. Fold change cut-off |log2(FC)| ≥ 2; FPKM cut-off ≥ 2 (at least three samples); FDR ≤ 0.05 (*n* = 3). Overlapping gene lists with sequencing results of cells co-incubated with platelets revealed a common regulation of 24 genes in both cell lines (|log2(FC)| ≥ 1; FPKM ≥ 2, at least three samples). **B** Heatmap showing 24 differentially regulated genes in BxPC-3 cells. **C** Enriched pathways of upregulated genes in BxPC-3 co-incubated with platelets were identified using gene set enrichment analysis (GSEA, http://www.broadinstitute.org/gsea); *p ≤ 0.05, **p ≤ 0.01, ***p ≤ 0.001). **D** STRING analysis (https://string-db.org) revealed protein-protein interactions between regulated genes based on experiments, databases, co-expressions and co-occurrence.

To analyze the signaling pathways that are altered under low-attachment conditions and potentially relevant for anoikis regulation, we performed RNA sequencing analysis of pancreatic cancer cell lines that were highly sensitive to detachment, namely, BxPC-3 and SU.86.86 (Supplementary Tables 1 and 2). We found that, applying cut-offs for the fold change, FPKM value and FDR, 265 and 184 genes were differentially expressed between adherent and non-adherent BxPC-3 and SU.86.86 cells, respectively. A comparison of the gene expression changes in both cell lines revealed an overlapping set of 109 genes (Fig. 2A). Among these 109 genes, four genes were commonly upregulated, whereas 103 genes were commonly downregulated in detached cells. Two genes showed an opposite regulation in both cell lines. Overrepresentation analysis of these 103 commonly downregulated genes using the PANTHER classification system (pantherdb.org/about.jsp) revealed that pathways related to cell cycle regulation were most significantly altered (Supplementary Table 3). Next, we examined the effects of platelets on gene expression programs in detached cells, which might be responsible for the increased survival and anoikis resistance mediated by platelets. Therefore, RNA sequencing analysis of detached BxPC-3 and SU.86.86 cells co-incubated with platelets for 48 h was performed (Supplementary Tables 4 and 5). Bioinformatics analysis revealed that 133 and 135 genes were significantly upregulated by platelet co-incubation in BxPC-3 and SU.86.86 cells, respectively. Given our primary interest in identifying genes that exhibited reduced expression following detachment and subsequently increased expression upon platelet interaction, these two gene lists were overlapped. By overlaying the results from both cell lines, we identified a set of 24 genes downregulated in both, BxPC-3 and SU.86.86 cells after low-attachment conditions and upregulated by platelet co-incubation (Fig. 2A, B, Supplementary Fig. 2 A). Like the pathways altered in detached cells, gene set enrichment analysis (GSEA) revealed that pathways related to the cell cycle and E2F1 signaling were positively enriched in cells co-incubated with platelets, both in BxPC-3 (Fig. 2 C) and SU.86.86 cells (Supplementary Fig. 2B). Intriguingly, STRING network analysis of the 24 overlapping genes highlighted one prominent cluster of genes displaying strong protein-protein interactions (Fig. 2D). In addition, analysis of transcriptome data from human pancreatic cancer samples using the Bailey RNAseq dataset [15] revealed strong positive and highly significant pairwise expression correlations of these platelet-regulated genes (Supplementary Fig. 2 C; *p* < 0.0001 for all correlations except for EFEMP1).

### FOXM1 expression is downregulated in non-adherent cells and associated with anoikis

One important transcription factor that is downregulated after cellular detachment but upregulated by platelet co-incubation is forkhead box transcription factor M1 (FOXM1). FOXM1 is a regulator of cell cycle-associated genes, is essential for DNA replication and mitosis [23, 24] and is implicated in the major deregulated pathways after cellular detachment and platelet co-incubation, as shown by PANTHER pathway analysis and GSEA. Indeed, performing cell cycle analyses after FOXM1 knockdown in BxPC-3 cells confirmed its role in cell cycle progression (Supplementary Fig. 3 A). In addition, the expression of numerous other genes downregulated by cellular detachment and upregulated by platelet co-incubation including *KIF2C*,* KIF20A*,* KIF4A*,* CCNA2*,* CCNB2*,* CDC20*, and *BIRC5*, was previously shown to be controlled by FOXM1 [24–29].

**Fig. 3:**
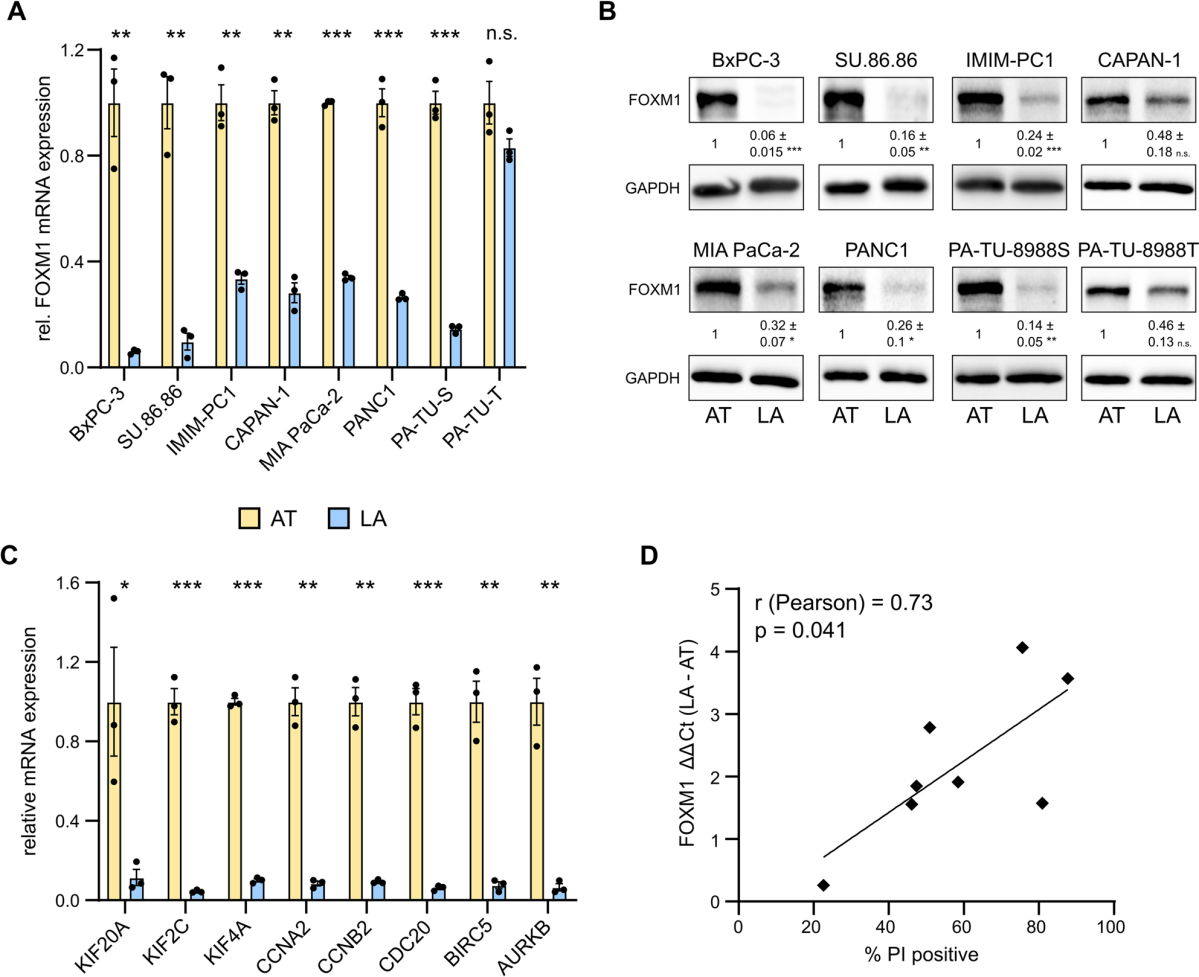
FOXM1 expression and the associated gene signature are upregulated in attached cells.** A** FOXM1 mRNA expression was significantly higher in attached (AT) versus low-attachment (LA) cultures in all tested cell lines except PA-TU-8988T cells. RPLP0 was used as a reference gene (BxPC-3: FC = 0.06 ± 0.005; Su.86.86: FC = 0.1 ± 0.032; IMIM-PC1: FC = 0.34 ± 0.021; CAPAN-1: FC = 0.28 ± 0.037; MIA Paca-2: FC = 0.34 ± 0.007; PANC-1: FC = 0.27 ± 0.007; PA-TU-8988 S: FC = 0.15 ± 0.009; PA-TU-8988T: FC = 0.83 ± 0.033). **B** Protein analysis and quantification of FOXM1 protein in attached and low-attachment cell cultures. GAPDH was used as a loading control. **C** Validation of RNA sequencing results of genes known to be regulated by FOXM1 in BxPC-3 cells using RT-qPCR. RPLP0 was used as a reference gene (*KIF20A*: FC = 0.12 ± 0.04; *KIF2C*: FC = 0.05 ± 0.004; *KIF4A*: FC = 0.10 ± 0.008; *CCNA2*: FC = 0.08 ± 0.012; *CCNB2*: FC = 0.09 ± 0.006; *CDC20*: FC = 0.06 ± 0.008; *BIRC5*: FC = 0.08 ± 0.017; *AURKB*: FC = 0.07 ± 0.018). **D** Changes in the expression of FOXM1 mRNA (RPLP0 normalized) were correlated with the percentage of PI-positive (= dead) cells under low-attachment. Bars and error bars represent the mean values and the corresponding SEMs (*n* = 3; *p ≤ 0.05, **p ≤ 0.01, ***p ≤ 0.001, n.s. = non-significant).

To confirm that FOXM1 RNA and protein expression are regulated by cellular detachment, RT-qPCR and western blot analyses were performed. With the exception of PA-TU-8988T, *FOXM1* mRNA was significantly downregulated more than 2-fold under low-attachment conditions in all pancreatic cancer cell lines tested (Fig. 3A). Similar expression changes in expression were observed at the protein level (Fig. 3B). In addition to FOXM1, RT-qPCR analyses confirmed attachment-dependent gene expression changes in FOXM1 downstream target genes in BxPC-3 (Fig. 3C) and SU.86.86 cells (Supplementary Fig. 3B). The genes were also regulated in IMIM-PC1 cells, which are highly sensitive to the low-attachment conditions (Supplementary Fig. 3 C). In contrast, the expression of potential FOXM1 target genes was not affected in PA-TU-8988T cells, which were resistant to detachment and showed only a weak deregulation of FOXM1 (Supplementary Fig. 3D). Finally, changes in *FOXM1* mRNA expression, depicted as the ∆∆Ct between adherent and non-adherent conditions, were significantly correlated with the percentage of PI-positive cells after 72 h of low-attachment (Fig. 3D), suggesting that FOXM1 downregulation is involved in anoikis-mediated cell death under low-attachment conditions.

### A FOXM1 expression signature is induced by platelets and is required for platelet-induced anoikis resistance

**Fig. 4:**
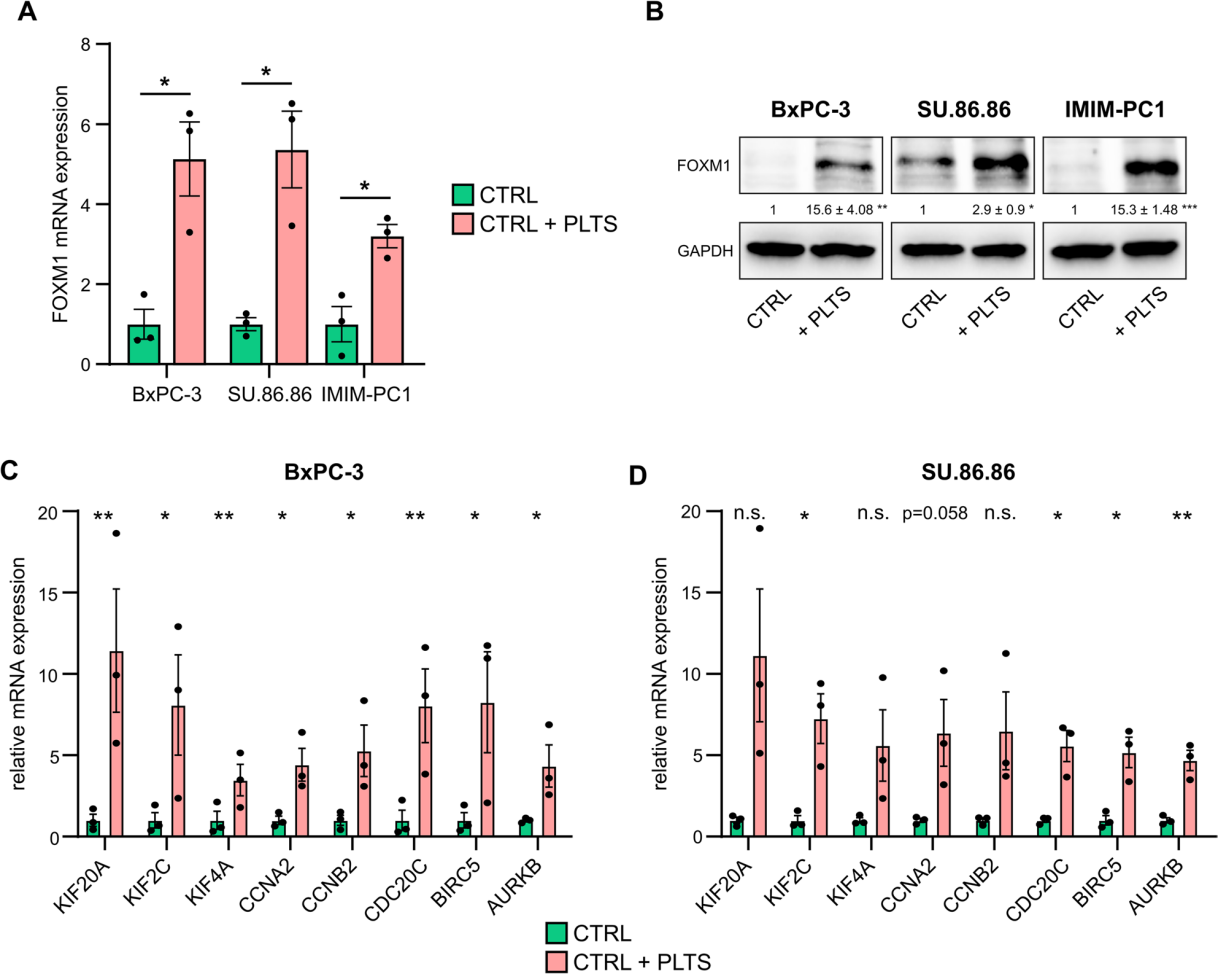
FOXM1 and its associated gene signature are regulated by platelet co-incubation. RT-qPCR analysis (**A**) and western blotting (**B**) showed increased protein expression of FOXM1 after platelet co-incubation in BxPC-3, SU.86.86 and IMIM-PC1 pancreatic cancer cell lines. RPLP0 was used as a reference gene for RT-qPCR. GAPDH was used as a loading control for western blotting. The mean intensity values and the corresponding SEMs compared with those of the controls are shown. **C**,** D** mRNA expression of FOXM1-regulated genes in BxPC-3 (C; *KIF2C*: FC = 8.09 ± 3.078; *KIF20A*: FC = 11.43 ± 3.797; *KIF4A*: FC = 4.42 ± 1.01; *CCNA2*: FC = 5.27 ± 1.586; *CCNB2*: FC = 3.47 ± 0.967; *CDC20*: FC = 8.04 ± 2.268; *BIRC5*: FC = 8.26 ± 3.099; *AURKB*: FC = 4.34 ± 1.297) and SU.86.86 (D; *KIF2C*: FC = 7.26 ± 1.525; *KIF20A*: FC = 11.14 ± 4.087; *KIF4A*: FC = 5.60 ± 2.197; *CCNA2*: FC = 6.37 ± 2.047; *CCNB2*: FC = 6.50 ± 2.393; *CDC20*: FC = 5.57 ± 0.961; *BIRC5*: FC = 5.18 ± 0.927; *AURKB*: FC = 4.68 ± 0.622) cells under low-attachment and co-incubation with platelets. Bars and error bars represent the mean values and the corresponding SEMs (*n* = 3; *p ≤ 0.05, **p ≤ 0.01, ***p ≤ 0.001, n.s. = non-significant)

To validate the RNA sequencing results and analyze the role of FOXM1 in platelet-induced anoikis resistance, we measured FOXM1 expression at the RNA and protein levels in BxPC-3, SU.86.86, and IMIM-PC1 cells that were co-incubated with platelets. RT-qPCR and western blot analyses confirmed a strong upregulation of *FOXM1* mRNA (Fig. 4A; BxPC-3: FC = 5.128 ± 0.926; SU.86.86: FC = 5.363 ± 0.959; IMIM-PC1: FC = 3.2 ± 0.292) and protein (Fig. 4B) by platelets in all three cancer cell lines after 48 h co-culture, whereas no FOXM1 protein expression was detected in murine platelets themselves (Supplementary Fig. 4A). In addition, we measured the changes in the mRNA expression of potential FOXM1 target genes in BxPC-3 (Fig. 4C), SU.86.86 (Fig. 4D) and IMIM-PC1 cells (Supplementary Fig. 4B) that were co-incubated with platelets for 48 h. Platelet co-culture consistently upregulated FOXM1 target genes across all three cell lines. In contrast, analysis of the anoikis-resistant cell line PA-TU-8988T revealed no significant changes in FOXM1 or FOXM1 target gene expression (Supplementary Fig. 4 C, D). To exclude species-specific effects, we co-incubated BxPC-3 and SU.86.86 cells with human platelets and observed results similar to those obtained with mouse platelets. Specifically, we detected increased FOXM1 protein (Supplementary Fig. 5 A, D) and mRNA (Supplementary Fig. 5B, E) expression, increased FOXM1 target gene expression (Supplementary Fig. 5B and E), and reduced anoikis rates after platelet co-incubation in both cell lines (Supplementary Fig. 5 C, F). Similarly, we could not detect FOXM1 protein expression in resting and activated human platelets (Supplementary Fig. 5G).

**Fig. 5:**
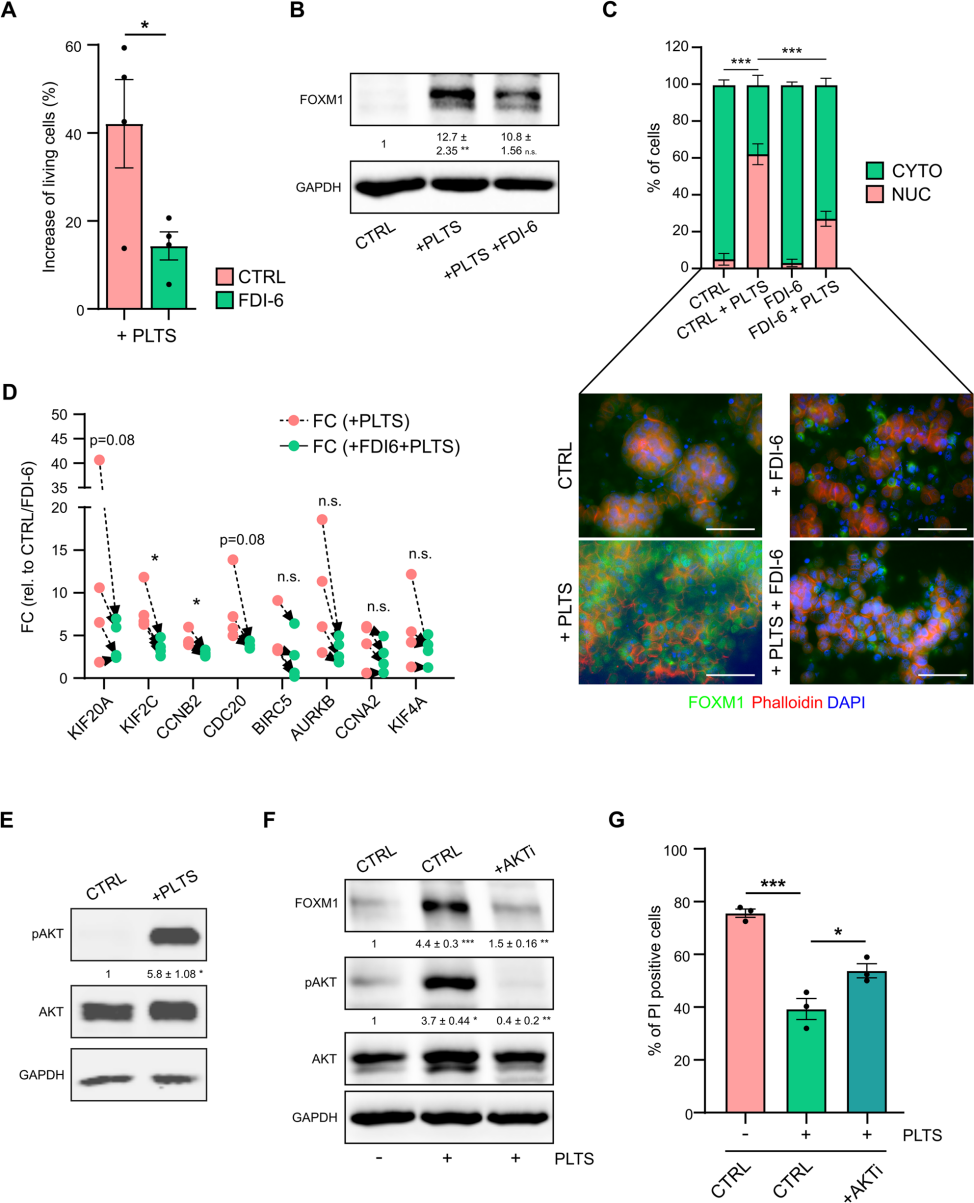
FOXM1 is important for platelet-mediated anoikis resistance and regulated by AKT. **A** Percentage (%) of increase in living (= PI-negative) BxPC-3 cells after platelet co-incubation in the presence or absence of the FOXM1 small molecule inhibitor FDI-6. **B** FOXM1 protein analysis and quantification after 48 h of platelet co-incubation with or without FDI-6 treatment. GAPDH was used as a loading control. **C** Immunofluorescence staining of FOXM1 protein and quantification of nuclear FOXM1 after platelet co-incubation with or without FDI-6 treatment. The actin cytoskeleton was visualized using phalloidin. Nuclear counterstaining was performed using DAPI. Scale bar = 50 μm. **D** Changes in gene expression after platelet co-incubation with or without FDI-6 (related to the untreated control or FDI-6 treatment), as measured via RT-qPCR. RPLP0 was used as a reference gene (*KIF20A*: FC = 14.91 ± 8.768 vs. FC = 4.51 ± 1.147; *KIF2C*: FC = 8.03 ± 1.283 vs. FC = 3.57 ± 0.470, *KIF4A*: FC = 5.78 ± 2.2995 vs. FC = 3.07 ± 0.627; *CCNA2*: FC = 4.15 ± 1.265 vs. FC = 2.55 ± 0.920; *CCNB2*: FC = 4.58 ± 0.470 vs. FC = 2.9 ± 0,165; *CDC20*: FC = 7.99 ± 2.016 vs. FC = 3.88 ± 0.183; *BIRC5*: FC = 4.76 ± 1.444 vs. FC = 2.48 ± 1.415, *AURKB*: FC = 9.73 ± 3.412 vs. FC = 3.33 ± 0.703). **E** Protein analysis and quantification of phosphorylated AKT (Ser473) versus total AKT in BxPC-3 cells after 2 h of platelet co-incubation. GAPDH was used as a loading control. **F** Protein analysis and quantification of phosphorylated AKT (Ser473) versus total AKT and FOXM1 protein after 48 h of platelet co-incubation in the presence or absence of the AKT inhibitor MK-2206. GAPDH was used as a loading control. **G** Percentage of PI-positive (= dead) cells after 72 h of low-attachment conditions with or without platelet co-incubation or the addition of MK-2206. Bars and error bars represent the mean values and the corresponding SEMs. For western blots, the mean intensity values and the corresponding SEMs relative to the control are shown (*n* = 3–4; *p ≤ 0.05, **p ≤ 0.01, ***p ≤ 0.001, n.s. = non-significant).

To validate whether FOXM1 is responsible for platelet-induced anoikis resistance, BxPC-3 cells were co-incubated with platelets in combination with or without FDI-6. FDI-6 is a highly specific FOXM1 inhibitor that displaces FOXM1 from its genomic targets, thereby interfering with its transcriptional program [30]. Although FDI-6 did not significantly affect the anoikis rate itself with the inhibitor concentration used (Supplementary Fig. 6 A), it significantly attenuated the platelet-induced anoikis resistance in BxPC-3 cells (Fig. 5A). While platelet co-incubation reduced the percentage of PI-positive cells by up to 60%, this effect was strongly inhibited by FDI-6, showing only a reduction of 5–20%. These findings suggest that the platelet-mediated increase in FOXM1 expression is an important driver of platelet-mediated anoikis resistance. Importantly, FDI-6 co-incubation only slightly reduced FOXM1 protein expression (Fig. 5B), confirming previous observations [30]. However, immunofluorescence analysis indicated that the nuclear localization of FOXM1 was induced by platelets and inhibited by the addition of FDI-6 (Fig. 5C). Consequently, treatment of detached BxPC-3 cells with FDI-6 impaired the platelet-induced upregulation of FOXM1 target genes involved in cell cycle regulation (Fig. 5D). To further validate the involvement of FOXM1 in platelet-induced anoikis resistance and exclude the off-target effects of FDI-6, we knocked down FOXM1 expression using two individual siRNAs (Supplementary Fig. 6B) before low-attachment culture. Again, platelet co-incubation reduced the number of dead BxPC-3 cells by > 50%. However, this effect was diminished by FOXM1 knockdown using both siRNAs (Supplementary Fig. 6 C). In addition, we inhibited FOXM1 using thiostrepton, an antibiotic and known FOXM1 inhibitor [31]. Like FDI-6 and FOXM1 siRNA transfection, thiostrepton interfered with the platelet effect and reduced the number of cells surviving under low-attachment culture conditions (Supplementary Fig. 6D). As published previously, we detected a slight downregulation of FOXM1 expression after thiostrepton treatment under steady-state conditions (Supplementary Fig. 6E, left panel). Moreover, thiostrepton inhibited the FOXM1 upregulation induced by platelets (Supplementary Fig. 6E, right panel). Similarly, stable overexpression of FOXM1 in BxPC-3 cells (Supplementary Fig. 6 F, G) decreased the number of PI-positive cells under low-attachment conditions by approximately 10% (Supplementary Fig. 6H).

Taken together, these data suggest that platelets enhance anoikis resistance by upregulating FOXM1, which induces a pro-survival gene expression program in cancer cells that could be targeted by modulating FOXM1 expression or function. In addition, preventing the platelet-mediated induction of FOXM1 might enhance anoikis and interfere with metastasis. Therefore, we ultimately wanted to understand how FOXM1 levels are induced in pancreatic cancer cells upon platelet co-culture. To evaluate whether juxtacrine or paracrine effects were responsible, we performed direct co-culture studies, as described above, as well as experiments in which BxPC-3 cells were exposed to the releasate of activated platelets. Interestingly, our data suggest that both FOXM1 upregulation (Supplementary Fig. 6I) and platelet-mediated anoikis resistance (Supplementary Fig. 6 J) are regulated via a direct interaction of platelets with cancer cells rather than by factors secreted by platelets. An evaluation of the phosphorylation status of 49 receptor tyrosine kinases revealed increased phosphorylation of EGFR and ERBB4, suggesting activation of ERBB signaling by platelets. In addition, increased phosphorylation of TIE2, EphA6, and EphA10 was detected (Supplementary Fig. 6 K). Notably, all of these receptors have been implicated in cancer and are associated with metastasis [32–36]; in particular, EGFR signaling has been linked to FOXM1 in other cancer types [37, 38]. One pathway commonly activated downstream of receptor tyrosine kinases is the PI3K-AKT signaling pathway [39, 40]. Importantly, previous studies have shown that FOXM1 expression can be regulated at the transcriptional and post-transcriptional level [41], and data from various solid tumors have demonstrated that FOXM1 expression is substantially controlled by the PI3K-AKT pathway [42]. Indeed, co-culture of BxPC-3 cells with platelets induced a fast and strong phosphorylation of AKT at serine 473 (Fig. 5E). Intriguingly, treatment with the AKT inhibitor MK-2206 prevented both the phosphorylation of AKT and FOXM1 upregulation upon platelet co-incubation (Fig. 5F). In addition, AKT inhibition was associated with the attenuation of platelet-mediated resistance to anoikis (Fig. 5G), suggesting that AKT, which is upstream of FOXM1, contributes to platelet-induced anoikis resistance. Overall, these data demonstrate that direct interactions between platelets and cancer cells induce FOXM1 expression after cellular detachment, which is regulated by AKT pathway activation. Consequently, the inhibition of AKT or FOXM1 increases anoikis rates and might represent a promising strategy to prevent metastasis in pancreatic cancer patients.

### FOXM1 expression is associated with survival and metastasis in PDAC

**Fig. 6:**
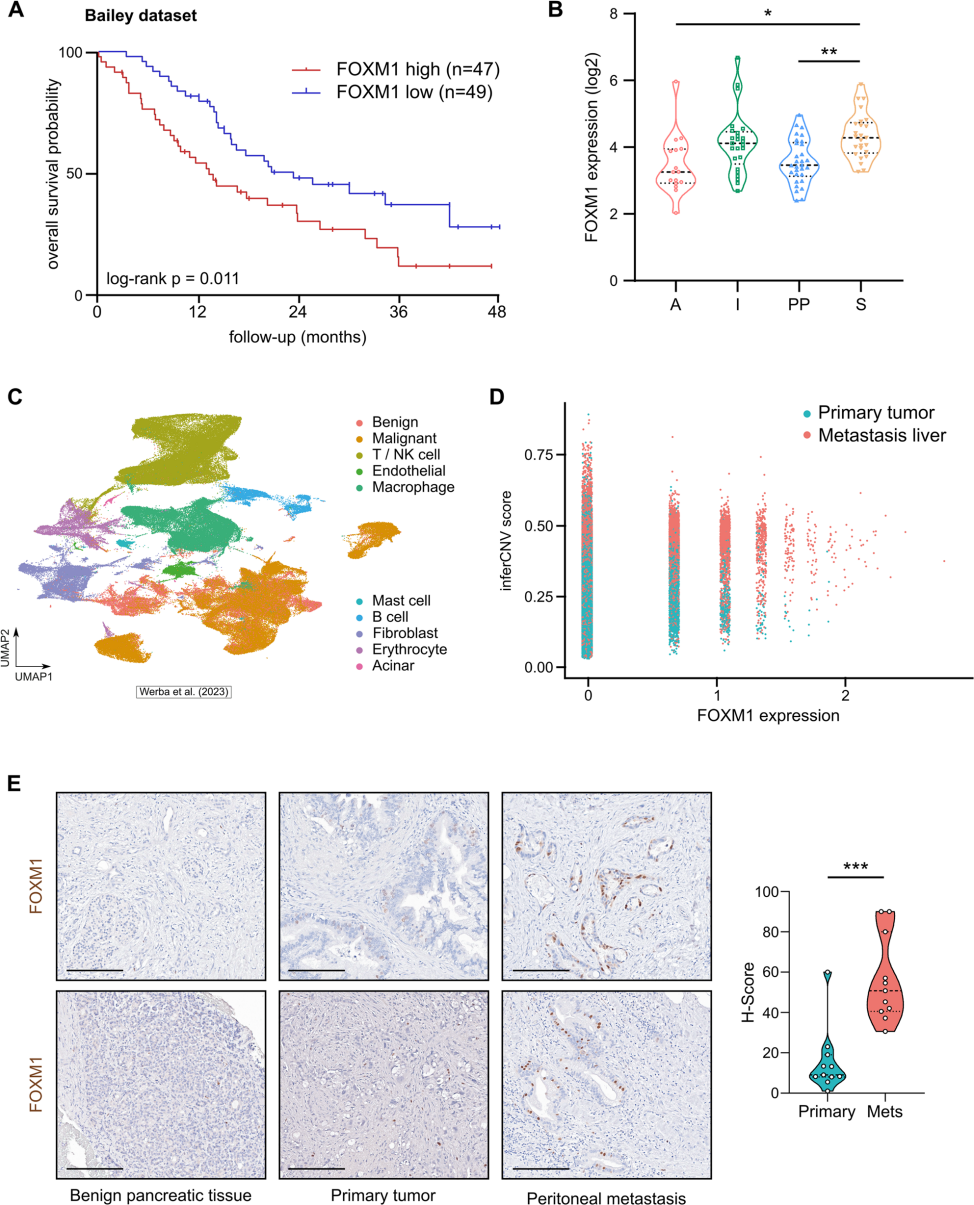
FOXM1 is crucial for patient prognosis and metastasis in PDAC. **A** Overall survival analysis for PDAC patients with low *FOXM1* (blue line, *n* = 49) versus high *FOXM1* (red line, *n* = 47) mRNA expression (Bailey dataset, data accessed via https://r2.amc.nl, log rank test). **B** Analysis of the Bailey PDAC dataset revealed significant upregulation of *FOXM1* mRNA expression in the squamous (S) subtype compared with the pancreatic progenitor (PP), and ADEX (A) subtypes (*p ≤ 0.05, **p ≤ 0.01). **C** UMAP plot depicting cell type composition of scRNA-seq PDAC samples. **D** FOXM1 expression levels in single epithelial cells from liver metastases and primary tumors, with high inferCNVscore indicative for malignant and low for benign epithelial cells. **E** Immunohistochemistry analysis of FOXM1 in eleven primary PDAC samples and matched peritoneal metastases. Quantification was performed using the histoscore (H-score), which is based on the percentage of positive cells (0–100%) multiplied by the staining intensity (0–3). Scale bar = 200 μm.

In line with our findings, FOXM1 has been suggested to be a master regulator of tumor metastasis [43]. FOXM1 is highly expressed in several cancers and is associated with poor survival [41]. Recently, it has been shown that the metastatic spread of ovarian cancer in the peritoneal cavity and ovarian cancer stemness are dependent on FOXM1 [37, 44]. Re-analysis of PDAC RNASeq datasets from Bailey et al. [15] revealed that FOXM1 expression was associated with worse overall survival (Fig. 6A). Moreover, FOXM1 is highly expressed in the most aggressive, i.e. squamous, subtype of pancreatic cancer (Fig. 6B). Similar results regarding overall survival probability were obtained when the TCGA dataset was explored [16] (log-rank *p* = 7.37e-04). When the survival data for the direct FOXM1 interactors identified in the network shown in Fig. 2D were re-analyzed, we found that only *KIF20A* and *CCNA2* presented a positive and statistically significant correlations with patient survival (*KIF20A*: *p* = 0.028; *CCNA2*: *p* = 9.58e-03), whereas for the other remaining FOXM1 target genes, no significant associations were detected (*AURKB*: *p* = 0.104; *KIF2C*: *p* = 0.424; *KIF4A*: *p* = 0.323; *CCNB2*: *p* = 0.053; *CDC20*: *p* = 0.197; *BIRC5*: *p* = 0.172). These findings suggest that while some FOXM1-interacting genes may contribute to disease outcomes, FOXM1 itself appears to play a central regulatory role in driving the coordinated expression of multiple downstream effectors that influence pancreatic cancer progression and patient prognosis. To further confirm the role of FOXM1 expression in the metastatic process of PDAC, we analyzed the transcriptome dynamics of FOXM1 in scRNA-seq data from patient samples from Werba et al. [17]. We inferred copy number variations (CNVs) to classify epithelial cells as benign or malignant (Fig. 6C) and juxtaposed FOXM1 expression in malignant cells of primary tumors and liver metastases (Fig. 6D). Intriguingly, we found that FOXM1 expression was significantly higher in malignant epithelial cells from liver metastases than in those from primary tumors (log2(FC) = 2.65, p_adj_ <2.23e-308). In addition, we found that FOXM1 is primarily expressed in tumor cells compared to normal epithelial cells (log2FC = 1.67, p_adj_=8.17e-179) and other cells of the tumor microenvironment, including fibroblasts (log2FC = 3.39, p_adj_=8.61e-257), different populations of immune cells (macrophages: log2FC = 2.6, p_adj_<2.23e-308; mast cells: log2FC = 3.69, p_adj_=4.87e-23; B cells: log2FC = 1.94, p_adj_=6.27e-60; T/NK cells: log2FC = 2.75, p_adj_<2.23e-308), and endothelial cells (log2FC = 3.19, p_adj_=2.48e-63). Consistent with the single-cell RNA sequencing data, immunohistochemical analysis of primary PDAC samples and peritoneal metastases revealed that FOXM1 expression was markedly elevated in metastatic PDAC lesions compared with the corresponding primary tumors from the same individuals, while no or only very weak staining was detected in benign pancreatic tissue (Fig. 6D). These findings underscore the translational importance of FOXM1 upregulation in PDAC and highlight FOXM1 as an important contributor to PDAC metastasis. Hence, targeting FOXM1 may represent a novel therapeutic strategy, particularly for metastatic PDAC.

## Discussion

Metastasis is the most lethal manifestation of cancer, and most cancer patients die because of metastatic disease [45]. In particular, patients with pancreatic cancer have a very poor overall survival rate after the disease has spread to distant organs [46]. A prerequisite for metastasis formation is the detachment of tumor cells from the primary tumor, crossing of the endothelial cell barrier, and survival in the bloodstream despite immune cell attack [3]. Here, we provide evidence that platelets activate survival pathways within detached pancreatic cancer cells, which are responsible for anoikis resistance. This finding is consistent with earlier reports showing that platelets closely interact with circulating tumor cells to promote their extravasation [47] and foster their survival in the peritoneal cavity [6, 48]. Their interactions are reciprocal. Cancer cells can activate platelets via the secretion of ADP [49], which leads to platelet degranulation and the secretion of pro-angiogenic, pro-tumorigenic and immunomodulatory factors [50, 51]. In turn, platelets induce cancer cell proliferation and facilitate metastasis by inducing a mesenchymal-like phenotype in cancer cells [7], activating a YAP1-dependent transcriptional program [6], or guiding the formation of an early metastatic niche [52]. Interestingly, a recent study showed that regular aspirin use decreased the risk of PDAC, particularly in patients with diabetes mellitus [53]. Another retrospective study reported that perioperative aspirin use was associated with significantly better overall survival in pancreatic cancer patients owing to an extended metastasis-free interval [54]. Platelets are likely the primary target of aspirin’s ability to inhibit platelet cyclooxygenase-1 [55]. Despite evidence supporting the role of platelets in tumor metastasis, prospective clinical trials have been scarce because anti-platelet medications may impair normal platelet function, resulting in bleeding complications. Here, we show that platelets interacting with detached pancreatic cancer cells induce a FOXM1-dependent transcriptional program that enhances cancer cell survival and promotes anoikis resistance. RNA sequencing analysis revealed that a set of 24 cell cycle-related genes consistently were upregulated in a panel of pancreatic cancer cells upon platelet interaction. FOXM1 is a well-known transcription factor that is upregulated in various human cancers [56–58]. In pancreatic cancer, FOXM1 is most abundant in the squamous subtype according to the RNASeq dataset of Bailey et al. [15], and its high expression is associated with worse survival. Interestingly, a genome-wide CRISPR-Cas9 knockout screen identified MYBL2 and its interaction with FOXM1 as critical drivers of PDAC metastasis [59]. Other studies confirmed the importance of FOXM1 in pancreatic cancer development and progression [60, 61]. However, the association between FOXM1 and anoikis resistance in pancreatic cancer cells has not yet been evaluated. Here, we show that *FOXM1* mRNA and protein expression is significantly downregulated in pancreatic cancer cells after detachment, and that the extent of this downregulation is inversely correlated with cell survival under low-attachment conditions. Co-culture of detached pancreatic cancer cells with murine and human platelets significantly increased the survival capacity of cancer cells and upregulated FOXM1 expression, as well as the expression of FOXM1 target genes. This likely occurs via direct platelet-cancer cell interaction and the activation of tyrosine kinase receptor signaling involving AKT activation, as we found a fast and durable phosphorylation of AKT protein kinase after platelet co-incubation and downregulation of FOXM1 expression after AKT inhibition. Inhibition of AKT with MK-2206, a selective pan-AKT inhibitor, confirmed its importance in the platelet-mediated survival of pancreatic cancer cells. These findings are in line with those of a previous study showing that FOXM1 is tightly regulated by the PI3K/AKT pathway in various cancers [42]. Moreover, the protein kinase B/AKT cellular pathway is suggested to be a key mediator of survival under low-attachment conditions [62]. Inhibition of FOXM1 using the small molecule inhibitor FDI-6 diminished platelet-induced anoikis resistance. Similar results were obtained following siRNA-mediated FOXM1 downregulation and FOXM1 inhibition using thiostrepton. In addition, FDI-6 treatment reduced the expression of FOXM1 transcriptional targets that were upregulated upon platelet co-incubation. Since both FOXM1 and its transcriptional targets play key roles in cell cycle control, our findings indicate that the disruption of cell cycle processes may contribute to anoikis resistance. Notably, integrated genomic and transcriptomic analyses of pancreatic cancer tissues have revealed that cell cycle progression is increased in metastases compared to primary pancreatic carcinomas [63]. By re-analyzing publicly available scRNA-seq datasets [17], we investigated the expression dynamics of FOXM1 in primary PDAC and liver metastasis samples. These analyses showed that FOXM1 is expressed primarily in tumor cells, suggesting that FOXM1 mainly regulates tumor cell intrinsic signaling pathways. This is confirmed by an analysis of quantitative transcriptomic data in several tumor types [64] and consistent with our immunohistochemical analysis, which revealed high nuclear FOXM1 expression in cancer cells compared with that in normal pancreas and surrounding tumor stroma. In addition, we detected significantly higher FOXM1 transcript levels in cancer cells from liver metastases than in those from primary tumors. This finding is in accordance with our immunohistochemical analyses showing elevated FOXM1 expression in peritoneal metastases compared with primary PDAC and with another study that revealed enrichment of tumorigenesis-relevant processes, including the FOXM1 signaling pathway, in metastatic lesions [65]. In addition, re-evaluation of RNA sequencing data from CTCs from metastatic PDAC patients revealed upregulation of FOXM1 and FOXM1 target genes in circulating cancer cells [66]. Therefore, targeting FOXM1 or its transcriptional targets may benefit patients with metastatic PDAC. Interestingly, the FOXM1 inhibitor thiostrepton is currently being evaluated in a phase II clinical trial to assess its safety and efficacy in treating patients with malignant pleural effusions (NCT05278975). Moreover, other clinical trials have targeted FOXM1 target genes, including Survivin (NCT06524063, NCT00108875) and AURKB/C (NCT01118611).

In conclusion, our in vitro experiments demonstrated that platelets increase FOXM1 expression in pancreatic cancer cells, which in turn contributes to platelet-mediated anoikis resistance. In addition, we provide evidence for elevated FOXM1 expression in metastatic lesions compared with primary PDAC lesions. In the future, a detailed analysis is necessary to determine the primary signal provided by platelets that is responsible for the observed FOXM1 upregulation and further to investigate the biological function of FOXM1 in vivo. These analyses could, in addition to direct targeting of FOXM1, lead to innovative approaches to limit metastasis formation by disrupting the platelet-tumor cell interaction in pancreatic cancer patients, particularly those with high platelet counts.

## Supplementary Information

Supplementary Material 1.

Supplementary Material 2

Supplementary Material 3

## Data Availability

The data underlying this article are available in the manuscript and in its online additional material. Additional materials generated are available from the corresponding author upon reasonable request.
