# Supplementary material for: FOXM1 regulates platelet-induced anoikis resistance in pancreatic cancer cells
Source: Cell Commun Signal. 2026 Jan 14;24:53. doi: 10.1186/s12964-025-02644-8 (PMC12849465; doi:10.1186/s12964-025-02644-8)
Supplement: Supplementary file 1 — Supplementary Material 1. [file 12964_2025_2644_MOESM1_ESM.pdf]

Supplementary Information

FOXM1 regulates platelet-induced anoikis resistance in pancreatic cancer cells

Alissa Ernesti-Soldatkin[1], Carolin T. Neu[1,#], Beate Heydel[1], Ferdinand Krannich[2], Helmut Laumen[2], Tony Gutschner[2], Monika Hämmerle[1,*]

[1]Institute of Pathology, Section of Experimental Pathology, Martin Luther University Halle-Wittenberg

[2]Department of Internal Medicine I, University Hospital Halle (Saale), Martin Luther University Halle-Wittenberg

[3]Institute of Molecular Medicine, Section for RNA Biology and Pathogenesis, Martin Luther University Halle-Wittenberg

[#] current address: Veterinary Faculty, Chair of Biochemistry and Chemistry, Ludwig Maximilian University Munich

* correspondence to: monika.haemmerle@uk-halle.de

Keywords: metastasis, platelet-cancer cell interaction, anoikis, FOXM1, pancreatic cancer

Supplementary Figure legends

Supplementary Figure 1: Anoikis rates change under low-attachment versus attached conditions in pancreatic cancer cells. (A-E) Pancreatic cancer cells were cultured under attached (AT) and low-attachment (LA) conditions for 72 hours, and the % of dead (= PI-positive) and living (= PI-negative) CAPAN-1 (A), MIA Paca-2 (B), PA-TU-8988S (C), and PA-TU-8988T (D) cells was measured using flow cytometry (upper bar graphs). In addition, increased apoptosis rates were confirmed using protein analysis and quantification of cleaved PARP (lower blots). GAPDH was used as a loading control. **(F)** PA-TU-8988T were co-incubated with 100 x 10^6 platelets (PLTS) and flow cytometry analysis, and western blotting was done as described above. Bars and error bars represent mean values and the corresponding SEMs. For western blots, mean intensity values and the corresponding SEMs are shown relative to controls (n = 3 - 4; *p ≤ 0.05, **p ≤ 0.01, ***p ≤ 0.001, n.s. = non-significant).

Supplementary Figure 2: GSEA analysis and heat map of differentially regulated genes in SU.86.86 cells. (A) Heat map of overlapping genes between attached (AT) and low-attachment (LA) condition and LA with or without platelet (PLTS) co-incubation. **(B)** Significantly enriched pathways in SU.86.86 cells as evaluated by gene set enrichment analysis (GSEA; p* ≤ 0.05, p*** ≤ 0.001). **(C)** Correlation analysis of 24 overlapping genes using transcriptome data of the Bailey dataset (expression data accessed via https://r2.amc.nl; p ≤ 0.0001 for all correlations except for EFEMP1).

Supplementary Figure 3: Impact of FOXM1 on cell cycle progression and qRT-PCR validation of RNA sequencing results. (A) Cell cycle profiles were analyzed in BxPC-3 cells after FOXM1 knockdown using two individual siRNAs. Bar graphs represent the % of cells in the subG1-, G1-, S- and G2-phase of the cell cycle. **(B-D)** mRNA expression of FOXM1-related genes after 48 hours of low-attachment (LA) or attachment (AT) in SU.86.86 (B), IMIM-PC1

(C) and PA-TU-8988T (D) cells. RPLP0 was used as a reference gene. Bars and error bars represent mean values and the corresponding SEMs (n = 3; *p ≤ 0.05, **p ≤ 0.01, ***p ≤ 0.001, n.s. = non-significant).

Supplementary Figure 4: FOXM1 expression in platelets and PA-TU-8988T cells and qRT-PCR validation of RNA sequencing results. (A) FOXM1 protein expression in attached BxPC-3 cells and in murine resting and activated platelets. GAPDH was used as a loading control. **(B, C)** mRNA expression of FOXM1-related genes in IMIM-PC1 (B) and PA-TU-8988T(C) cells after 48h of low-attachment with or without platelet (PLTS) co-incubation. RPLP0 was used as a reference gene. **(D)** FOXM1 protein expression in PA-TU-8988-T cells after 48h of low-attachment with or without platelets (PLTS) co-incubation. Bars and error bars represent mean values and the corresponding SEMs. For protein analysis, mean intensity values and the corresponding SEMs are shown relative to controls (n = 3; *p ≤ 0.05, **p ≤ 0.01, ***p ≤ 0.001, n.s. = non-significant).

Supplementary Figure 5: Impact of human platelets on FOXM1 and FOXM1 target gene expression and anoikis resistance. (A, D) Western blotting show increased protein expression of FOXM1 after co-incubation with human platelets in BxPC-3 (A) and SU.86.86 (D) cells. GAPDH was used as a loading control. **(B, E)** RT-qPCR analysis show increased FOXM1 and FOXM1 target gene mRNA expression in BxPC-3 (B) and SU.86.86 (E) cells. RPLP0 was used as a reference gene. **(C, F)** Flow cytometry analysis of dead (PI-positive) and living (PI-negative) BxPC-3 (C) and SU.86.86 (F) cells, shown in %, after 72 h co-incubation with human platelets. **(G)** FOXM1 protein expression in attached BxPC-3 cells and in human resting and activated platelets. GAPDH was used as a loading control. Bars and error bars represent mean values and the corresponding SEMs. For protein analysis, mean intensity values and the corresponding SEMs are shown relative to controls (n = 3; *p ≤ 0.05, **p ≤ 0.01, ***p ≤ 0.001, n.s. = non-significant).

Supplementary figure 6: Functional evaluation of FOXM1 in BxPC-3 cells. (A) Percentage (%) of PI-positive (= dead) cells after low-attachment BxPC-3 cultures in control conditions or with FDI-6 treatment. **(B)** FOXM1 expression in low-attachment BxPC-3 cultures with or without platelet co-incubation and siRNA transfection. RPLP0 was used as a reference gene. **(C)** Percentage (%) of increase of living (PI-negative cells after platelet co-incubation of BxPC-3 cells transfected with control siRNA or two individual FOXM1 siRNAs. **(D)** Percentage (%) of increase of living (PI-negative) BxPC-3 cells co-incubated with platelets with or without thiostrepton treatment. **(E)** FOXM1 protein expression under attached conditions (left panel) and after platelet co-incubation under low- attachment (right panel), both with or without thiostrepton treatment. GAPDH was used as a loading control. **(F)** FOXM1 mRNA and **(G)** protein expression in BxPC-3 cells with (FOXM1) or without (EV = empty vector) FOXM1 overexpression. RPLP0 was used as a reference gene and RPL7 was used as a loading control. **(H)** Percentage (%) of PI-positive (dead) cells after 72 hours of low-attachment BxPC-3 cultures with or without FOXM1 overexpression. **(I)** FOXM1 protein expression in BxPC-3 cells after direct platelet co-culture (+PLTS) and in BxPC-3 cells co-incubated with platelet releasate (+REL). GAPDH was used as a loading control. **(J)** Flow cytometry analysis of dead (PI-positive) and living (PI-negative) BxPC-3 cells after direct platelet co-culture (+PLTS) and in BxPC-3 cells co-incubated with platelet releasate (+REL). **(K)** Human phospho-RTK array of isolated BxPC-3 proteins with or without platelet co-culture or of isolated murine platelet proteins. Bars and error bars represent mean values and the corresponding SEMs. For western blots, mean intensity values and the corresponding SEMs are shown relative to controls (n = 3 - 4; *p ≤ 0.05, **p ≤ 0.01, ***p ≤ 0.001, n.s. = non-significant).

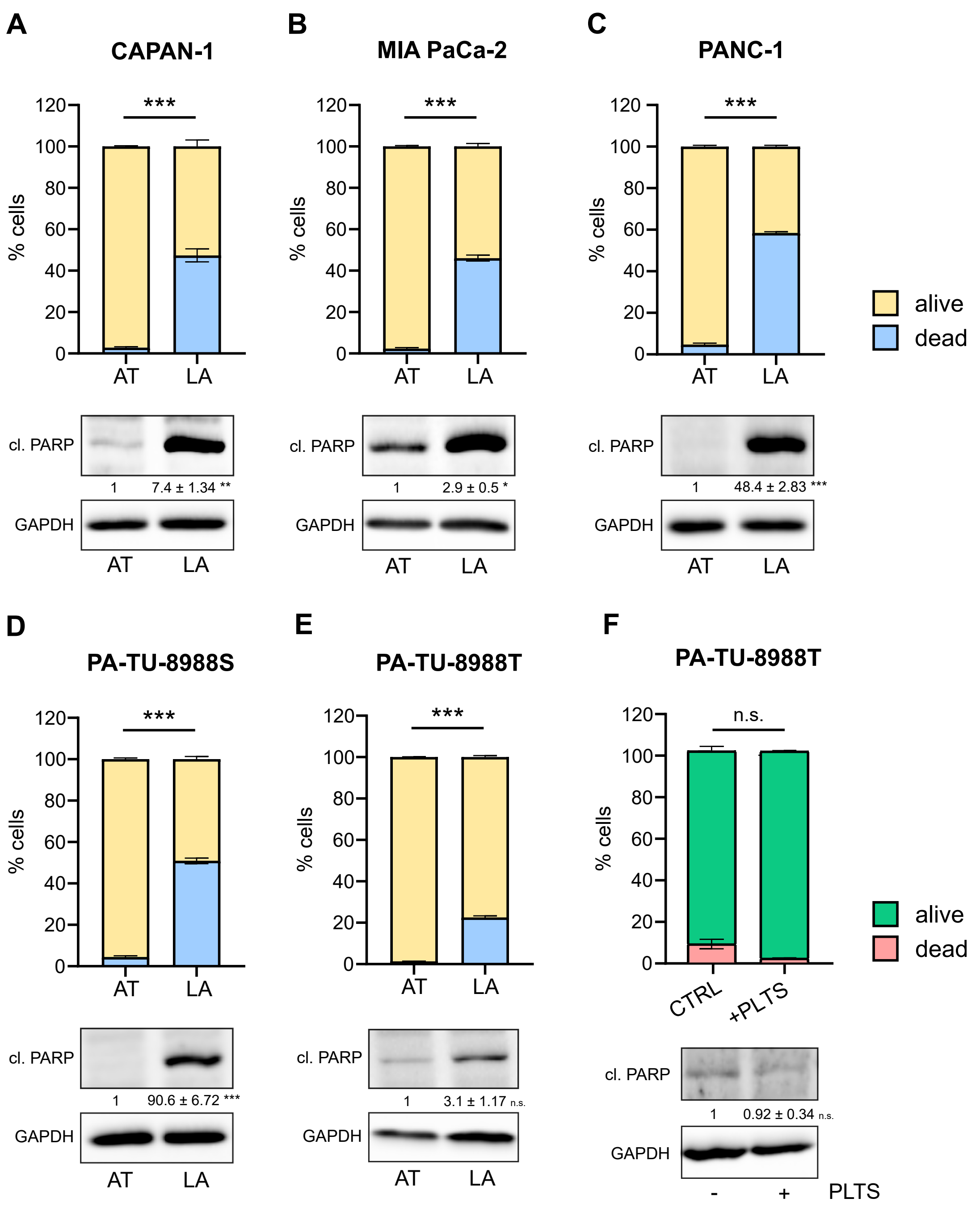

Supplementary Figure 1

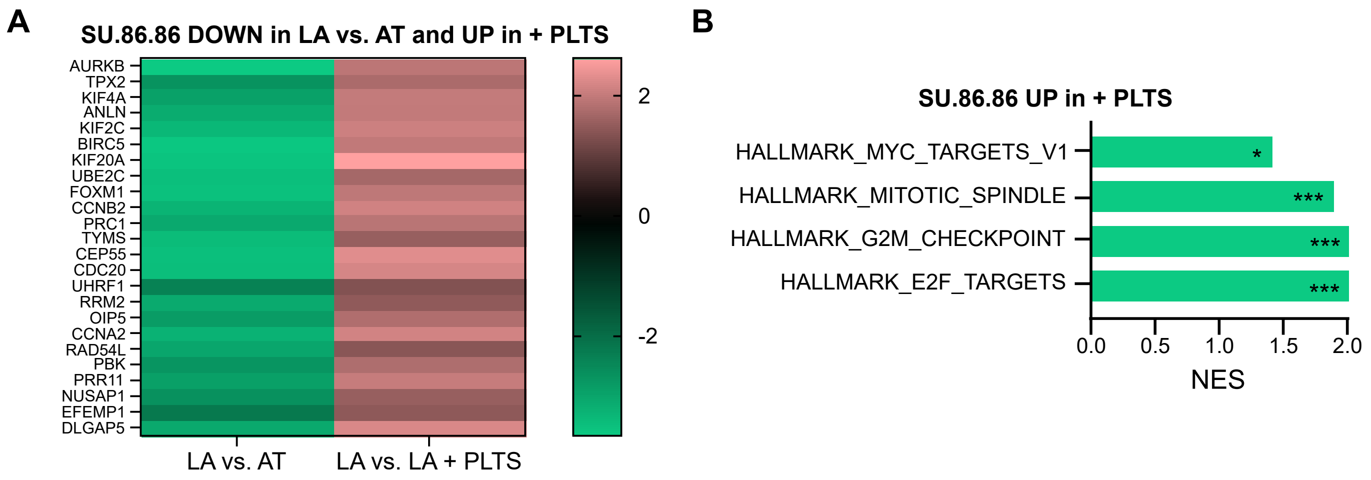

C

	FOXM1	KIF2C	KIF20A	KIF4A	CCNA2	CCNB2	CDC20	BIRC5	AURKB	TPX2	ANLN	UBE2C	DLGAP5	PRC1	TYMS	CEP55	RRM2	OIP5	RAD54L	PBK	PRR11	NUSAP1	EFEMP1
FOXM1	1.00	0.75	0.65	0.80	0.73	0.79	0.75	0.71	0.78	0.79	0.64	0.80	0.63	0.74	0.65	0.71	0.68	0.63	0.72	0.50	0.70	0.69	-0.06
KIF2C	0.75	1.00	0.80	0.82	0.86	0.77	0.95	0.78	0.76	0.84	0.75	0.83	0.81	0.79	0.64	0.70	0.75	0.70	0.80	0.66	0.68	0.76	-0.07
KIF20A	0.65	0.80	1.00	0.79	0.79	0.78	0.81	0.71	0.71	0.81	0.81	0.74	0.86	0.74	0.57	0.69	0.65	0.69	0.61	0.65	0.69	0.75	-0.02
KIF4A	0.80	0.82	0.79	1.00	0.83	0.84	0.81	0.77	0.81	0.88	0.80	0.84	0.81	0.78	0.60	0.77	0.79	0.72	0.71	0.61	0.68	0.80	-0.17
CCNA2	0.73	0.86	0.79	0.83	1.00	0.80	0.83	0.76	0.78	0.81	0.77	0.79	0.86	0.77	0.65	0.74	0.75	0.74	0.66	0.66	0.66	0.77	-0.10
CCNB2	0.79	0.77	0.78	0.84	0.80	1.00	0.82	0.80	0.77	0.82	0.75	0.80	0.76	0.87	0.55	0.72	0.71	0.85	0.67	0.63	0.68	0.88	-0.11
CDC20	0.75	0.95	0.81	0.81	0.83	0.82	1.00	0.81	0.75	0.82	0.75	0.84	0.80	0.80	0.65	0.73	0.73	0.72	0.79	0.64	0.67	0.76	-0.07
BIRC5	0.71	0.78	0.71	0.77	0.76	0.80	0.81	1.00	0.75	0.80	0.71	0.76	0.70	0.74	0.60	0.69	0.72	0.69	0.65	0.66	0.78	0.74	-0.02
AURKB	0.78	0.76	0.71	0.81	0.78	0.77	0.75	0.75	1.00	0.85	0.72	0.81	0.73	0.74	0.61	0.72	0.66	0.61	0.66	0.53	0.70	0.70	-0.04
TPX2	0.79	0.84	0.81	0.88	0.81	0.82	0.82	0.80	0.85	1.00	0.88	0.90	0.81	0.78	0.59	0.78	0.73	0.65	0.73	0.59	0.80	0.74	-0.07
ANLN	0.64	0.75	0.81	0.80	0.77	0.75	0.75	0.71	0.72	0.88	1.00	0.78	0.78	0.70	0.53	0.70	0.67	0.61	0.66	0.55	0.73	0.67	
UBE2C	0.80	0.83	0.74	0.84	0.79	0.80	0.84	0.76	0.81	0.90	0.78	1.00	0.79	0.76	0.58	0.81	0.67	0.63	0.72	0.51	0.72	0.69	-0.04
DLGAP5	0.63	0.81	0.86	0.81	0.86	0.76	0.80	0.70	0.73	0.81	0.78	0.79	1.00	0.74	0.54	0.69	0.68	0.66	0.60	0.65	0.65	0.71	-0.09
PRC1	0.74	0.79	0.74	0.78	0.77	0.87	0.80	0.74	0.74	0.78	0.70	0.76	0.74	1.00	0.57	0.77	0.71	0.80	0.67	0.66	0.59	0.88	-0.18
TYMS	0.65	0.64	0.57	0.60	0.65	0.55	0.65	0.60	0.61	0.59	0.53	0.58	0.54	0.57	1.00	0.55	0.60	0.57	0.55	0.45	0.49	0.60	0.06
CEP55	0.71	0.70	0.69	0.77	0.74	0.72	0.73	0.69	0.72	0.78	0.70	0.81	0.69	0.77	0.55	1.00	0.61	0.61	0.59	0.51	0.59	0.69	-0.07
RRM2	0.68	0.75	0.65	0.79	0.75	0.71	0.73	0.72	0.66	0.73	0.67	0.67	0.68	0.71	0.60	0.61	1.00	0.67	0.66	0.67	0.62	0.76	-0.08
OIP5	0.63	0.70	0.69	0.72	0.74	0.85	0.72	0.69	0.61	0.65	0.61	0.63	0.66	0.80	0.57	0.61	0.67	1.00	0.57	0.69	0.48	0.89	-0.09
RAD54L	0.72	0.80	0.61	0.71	0.66	0.67	0.79	0.65	0.66	0.73	0.66	0.72	0.60	0.67	0.55	0.59	0.66	0.57	1.00	0.50	0.61	0.63	-0.19
PBK	0.50	0.66	0.65	0.61	0.66	0.63	0.64	0.66	0.53	0.59	0.55	0.51	0.65	0.66	0.45	0.51	0.67	0.69	0.50	1.00	0.53	0.69	-0.04
PRR11	0.70	0.68	0.69	0.68	0.66	0.68	0.67	0.78	0.70	0.80	0.73	0.72	0.65	0.59	0.49	0.59	0.62	0.48	0.61	0.53	1.00	0.56	-0.07
NUSAP1	0.69	0.76	0.75	0.80	0.77	0.88	0.76	0.74	0.70	0.74	0.67	0.69	0.71	0.88	0.60	0.69	0.76	0.89	0.63	0.69	0.56	1.00	-0.14
EFEMP1	-0.06	-0.07	-0.02	-0.17	-0.10	-0.11	-0.07	-0.02	-0.04	-0.07		-0.04	-0.09	-0.18	0.06	-0.07	-0.08	-0.09	-0.19	-0.04	-0.07	-0.14	1.00

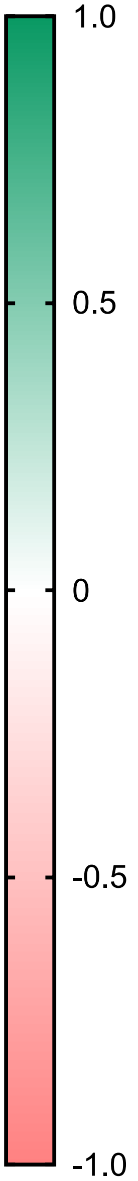

Supplementary Figure 2

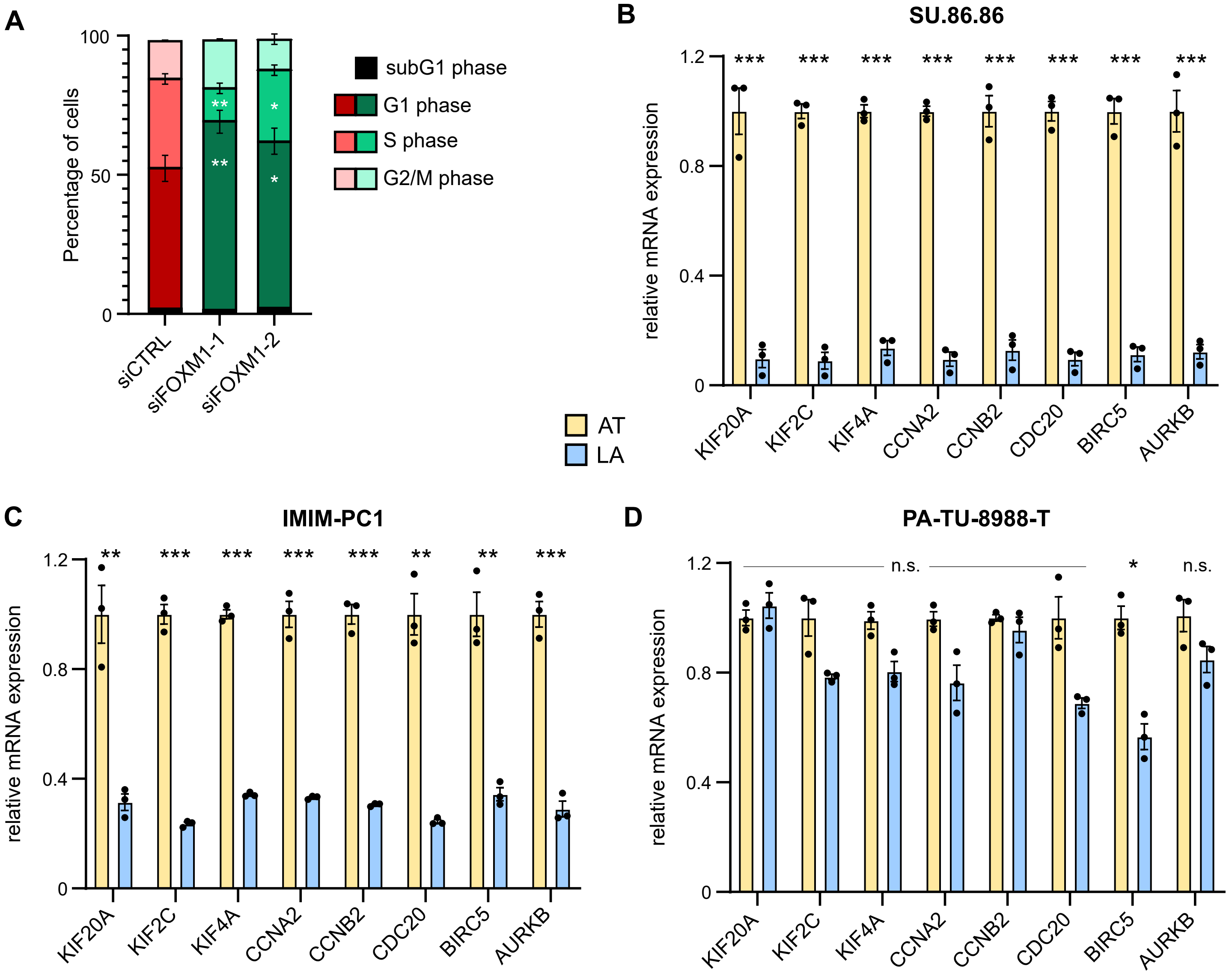

Supplementary Figure 3

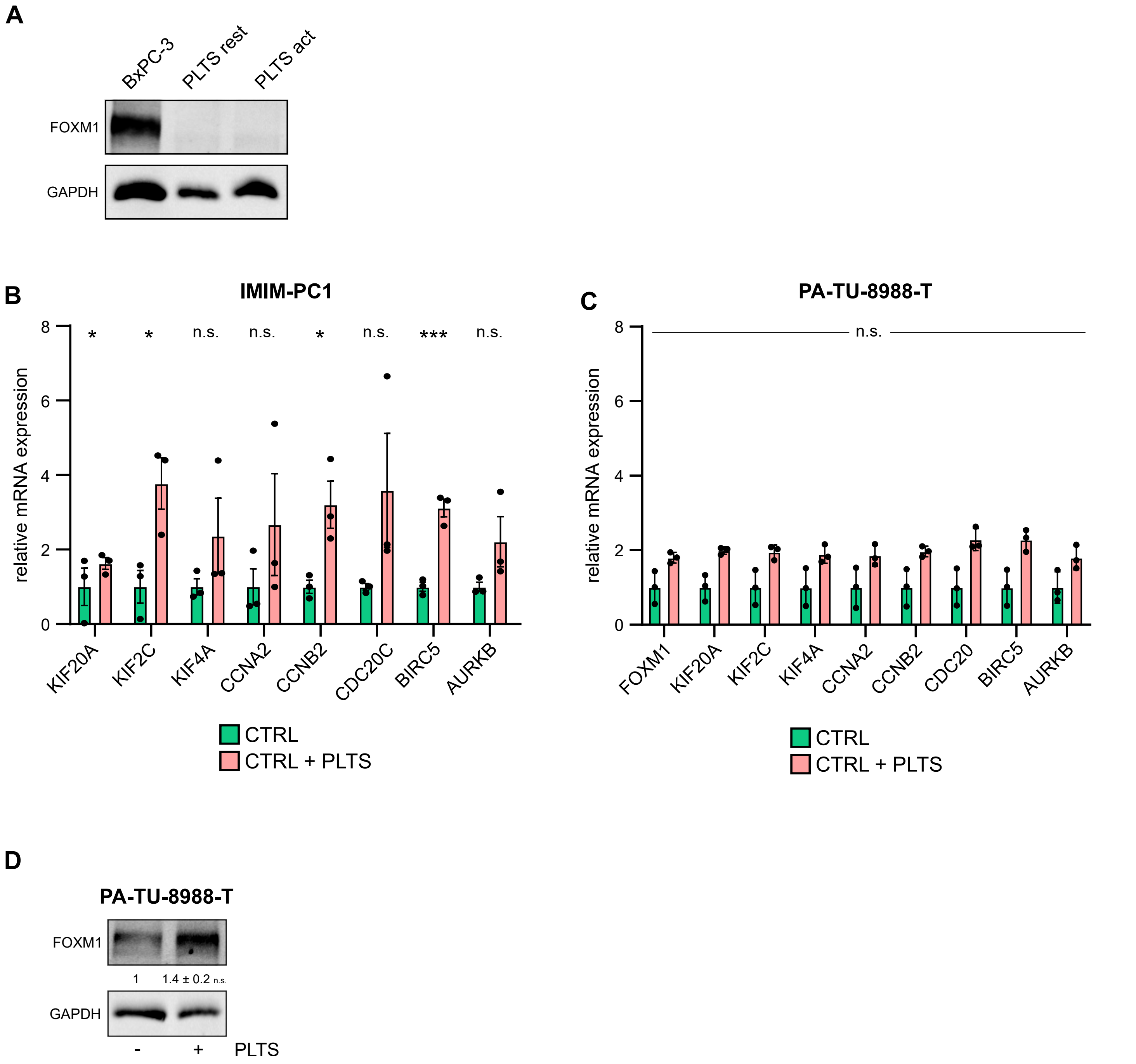

Supplementary Figure 4

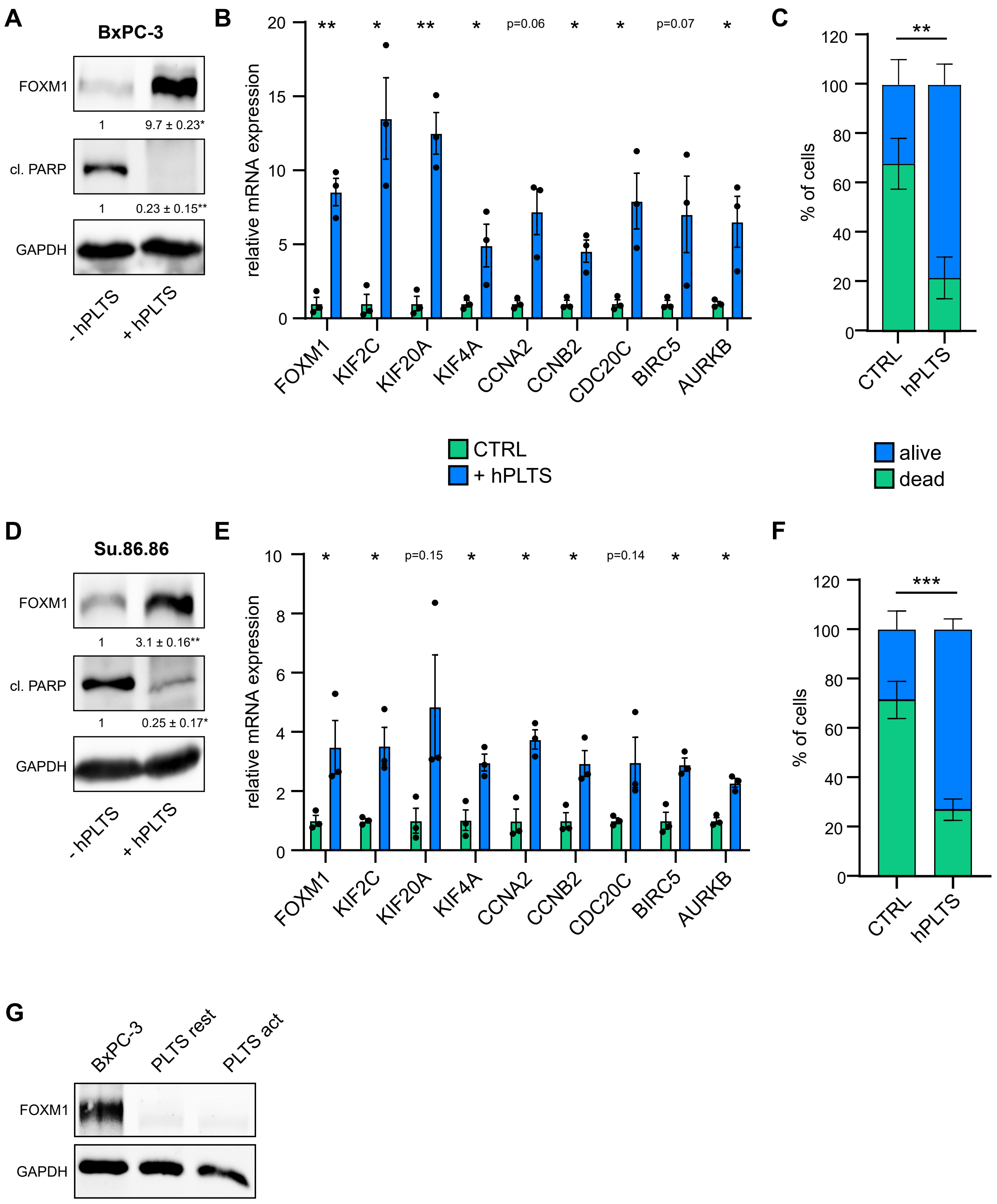

Supplementary Figure 5

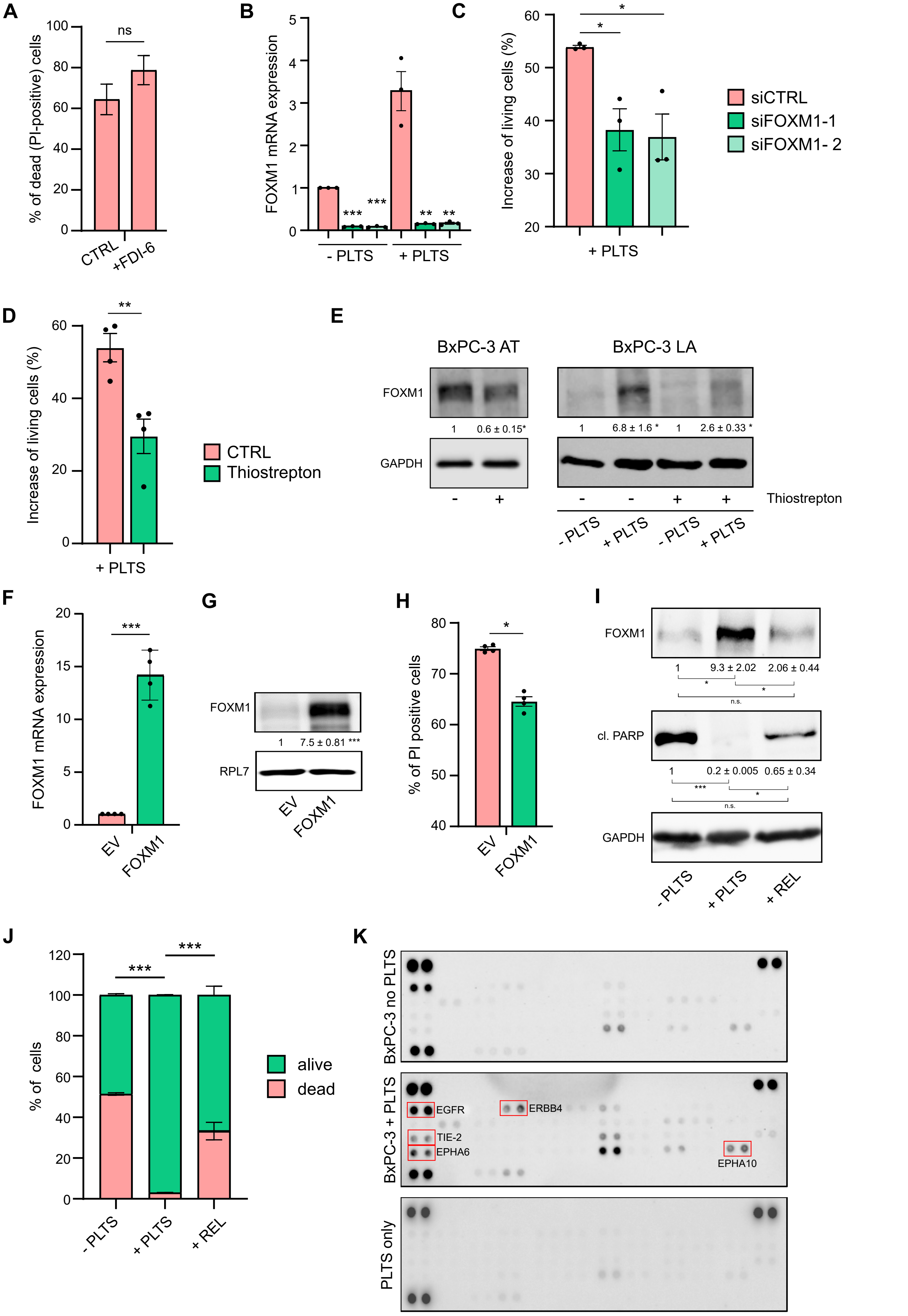

Supplementary Figure 6
