# Supplementary material for: FOXM1 regulates platelet-induced anoikis resistance in pancreatic cancer cells
Source: Cell Commun Signal. 2026 Jan 14;24:53. doi: 10.1186/s12964-025-02644-8 (PMC12849465; doi:10.1186/s12964-025-02644-8)

Figure 1

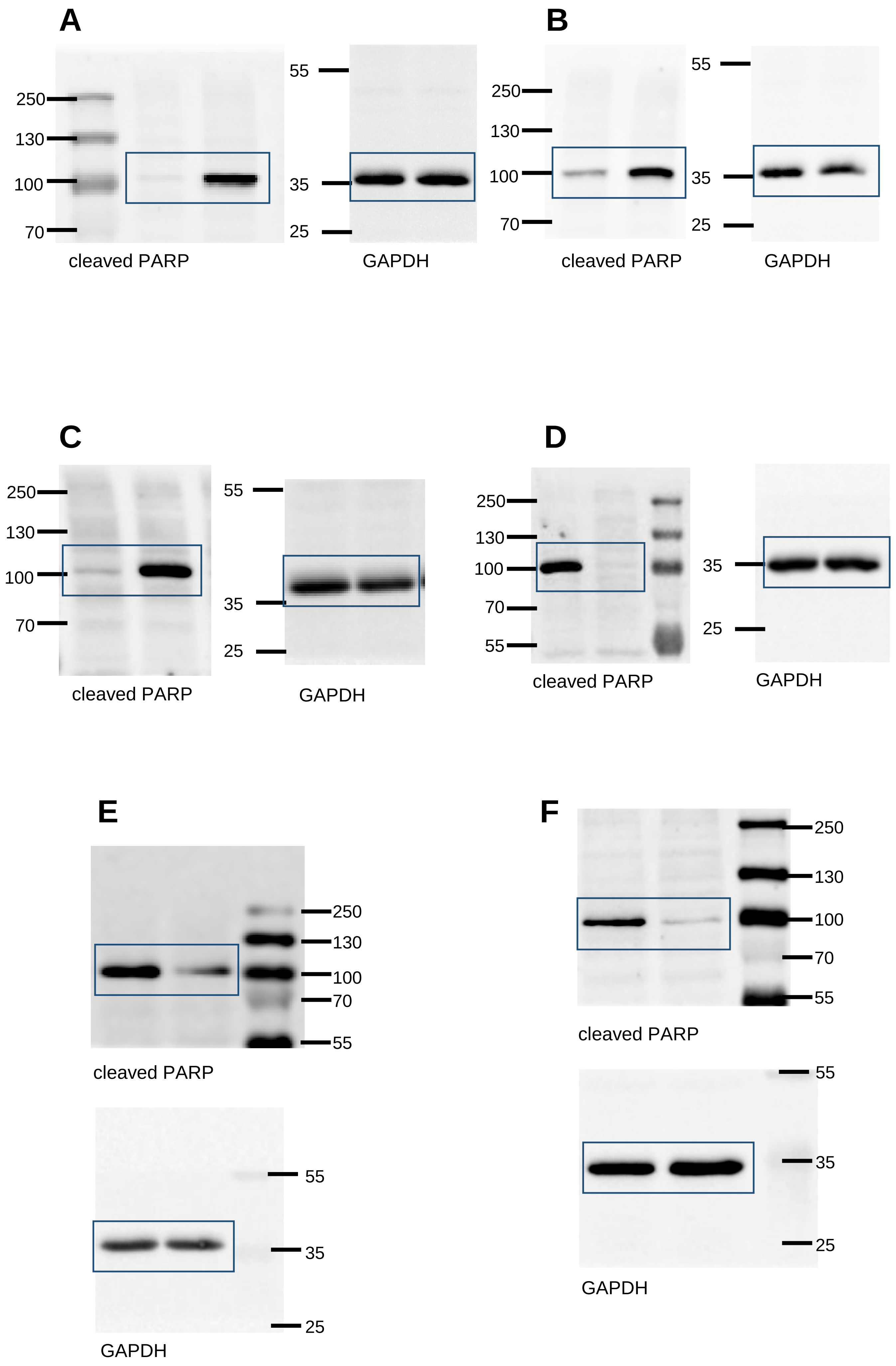
A
250
130
100
70
cleaved PARP
55
35
25
GAPDH
B
250
130
100
70
cleaved PARP
55
35
25
GAPDH
C
250
130
100
70
cleaved PARP
55
35
25
GAPDH
D
250
130
100
70
55
cleaved PARP
35
25
GAPDH
E
250
130
100
70
55
cleaved PARP
55
35
25
GAPDH
F
250
130
100
70
55
cleaved PARP
55
35
25
GAPDH

Figure 3B

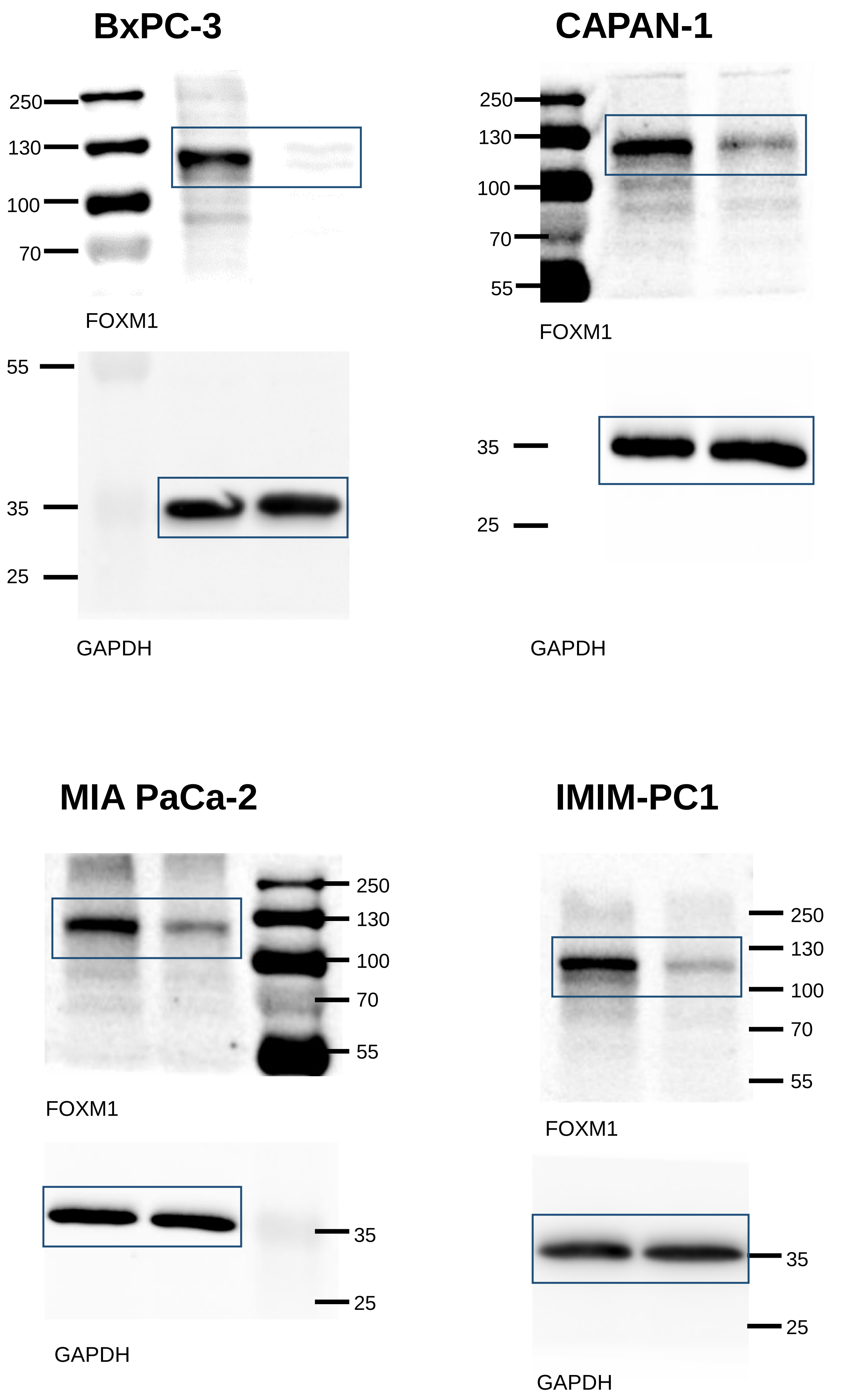
BxPC-3
250
130
100
70
FOXM1
55
35
25
GAPDH
CAPAN-1
250
130
100
70
55
FOXM1
35
25
GAPDH
MIA PaCa-2
250
130
100
70
55
FOXM1
35
25
GAPDH
IMIM-PC1
250
130
100
70
55
FOXM1
35
25
GAPDH

Figure 3B

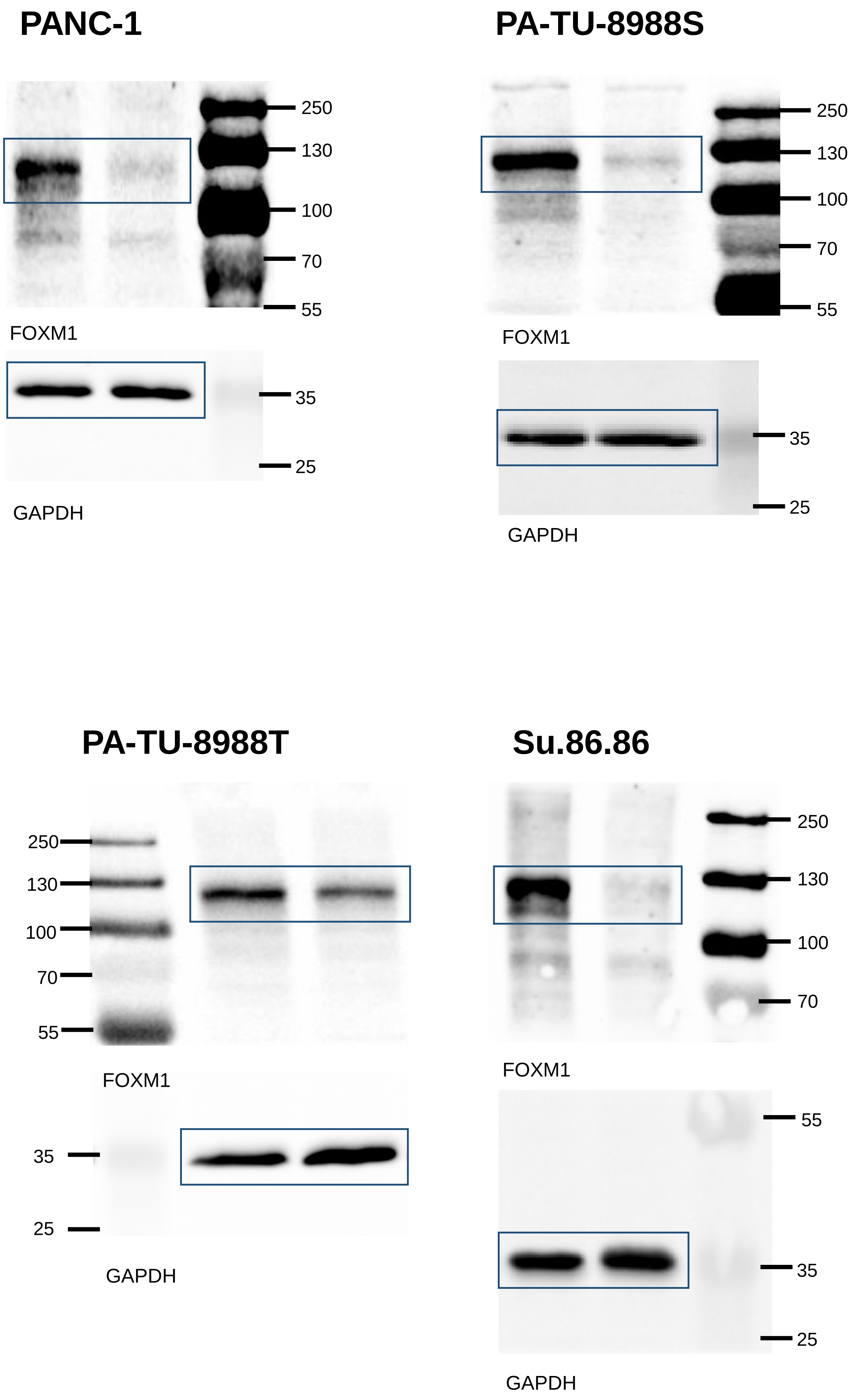
PANC-1
250
130
100
70
55
FOXM1
35
25
GAPDH
PA-TU-8988S
250
130
100
70
55
FOXM1
35
25
GAPDH
PA-TU-8988T
250
130
100
70
55
FOXM1
35
25
GAPDH
Su.86.86
250
130
100
70
FOXM1
55
35
25
GAPDH

Figure 4B

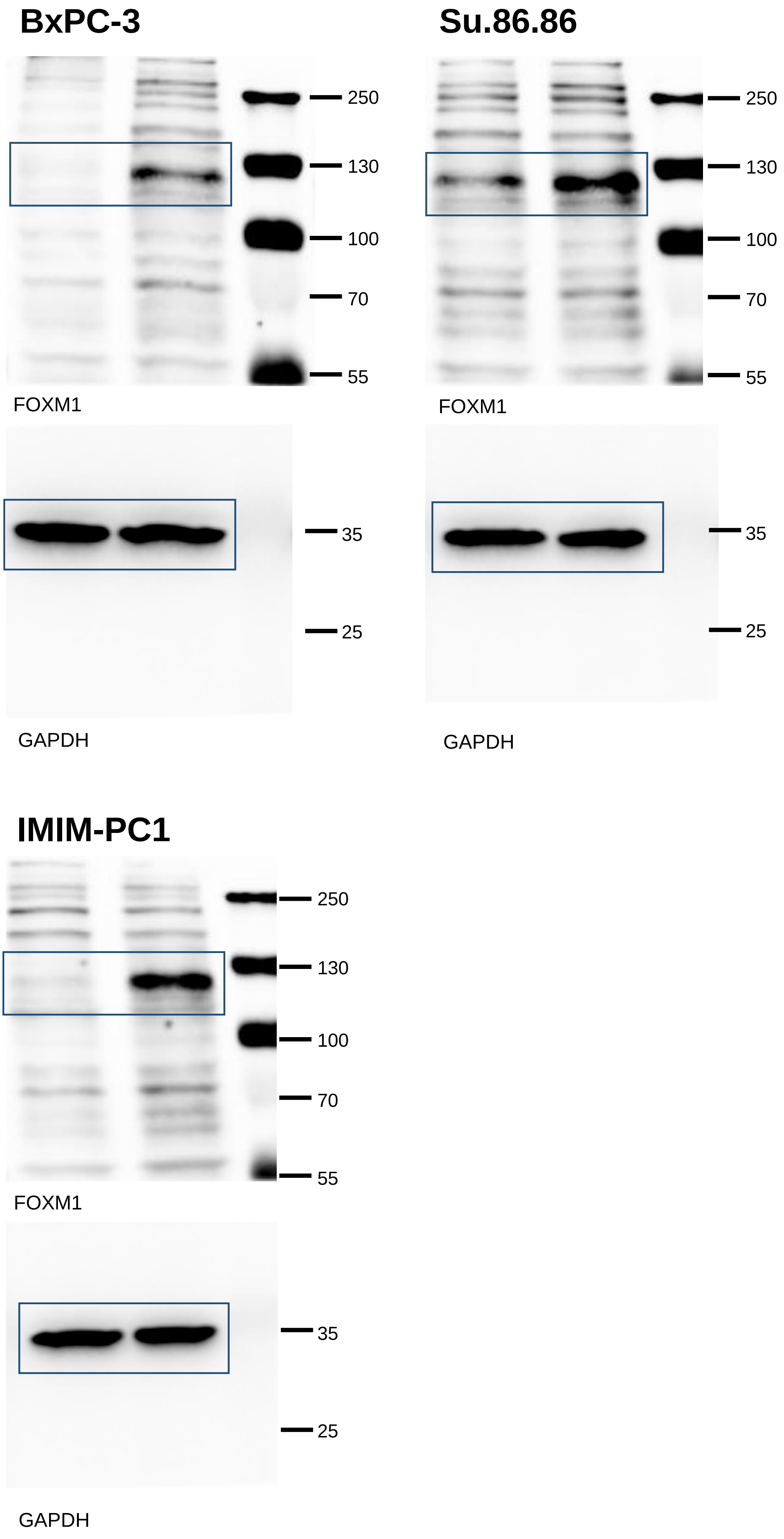
BxPC-3
250
130
100
70
55
FOXM1
35
25
GAPDH
Su.86.86
250
130
100
70
55
FOXM1
35
25
GAPDH
IMIM-PC1
250
130
100
70
55
FOXM1
35
25
GAPDH

Figure 5

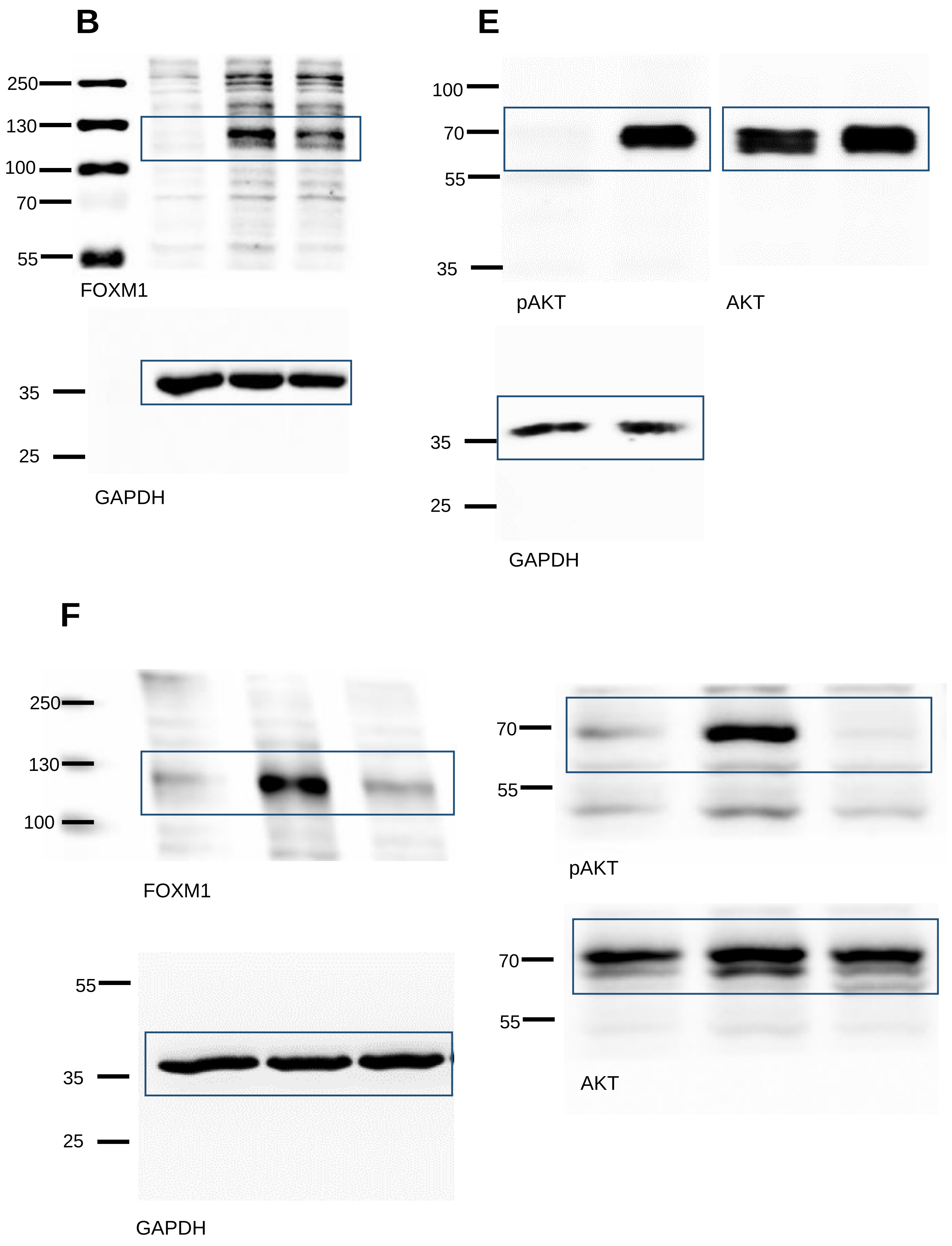
B
250
130
100
70
55
FOXM1
35
25
GAPDH
E
100
70
55
35
pAKT
AKT
35
25
GAPDH
F
250
130
100
FOXM1
70
55
pAKT
55
35
25
GAPDH
70
55
AKT

Supplementary Figure 1

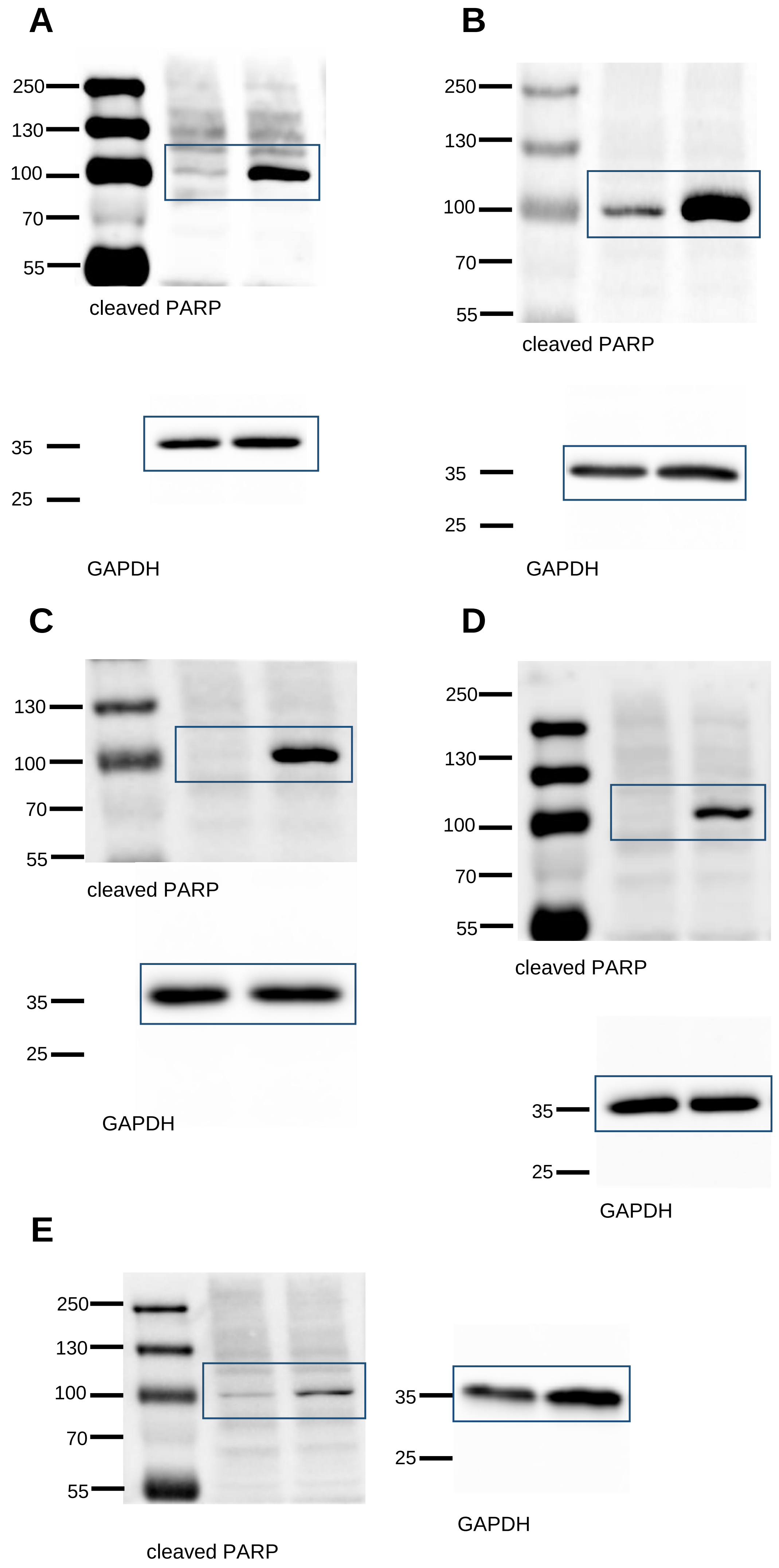

Supplementary Figure 1

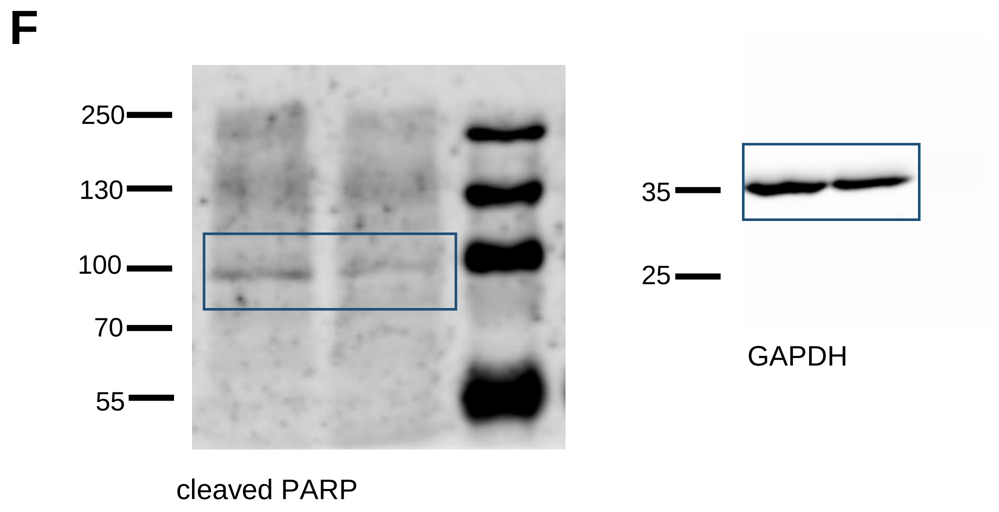

Supplementary Figure 4

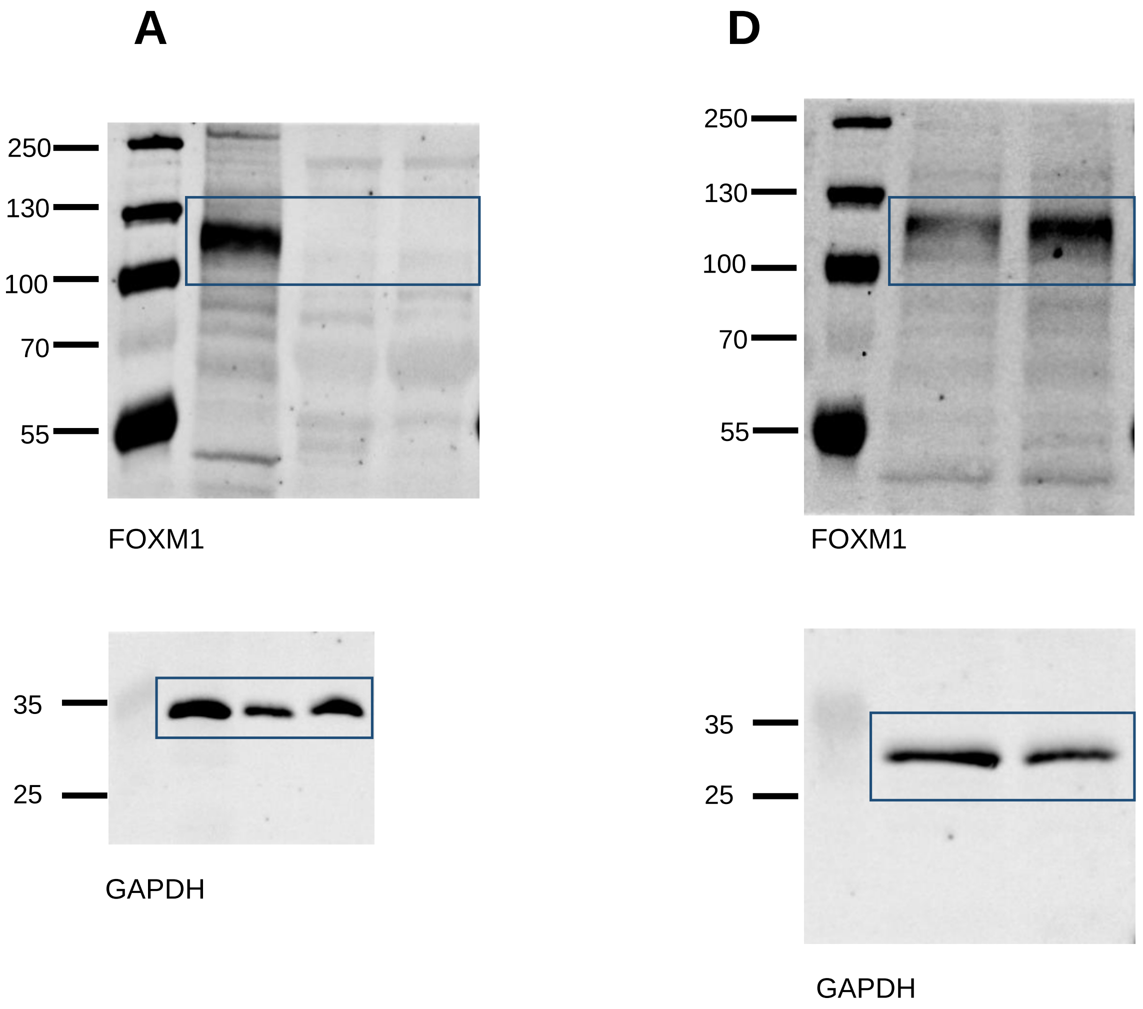

Supplementary Figure 5

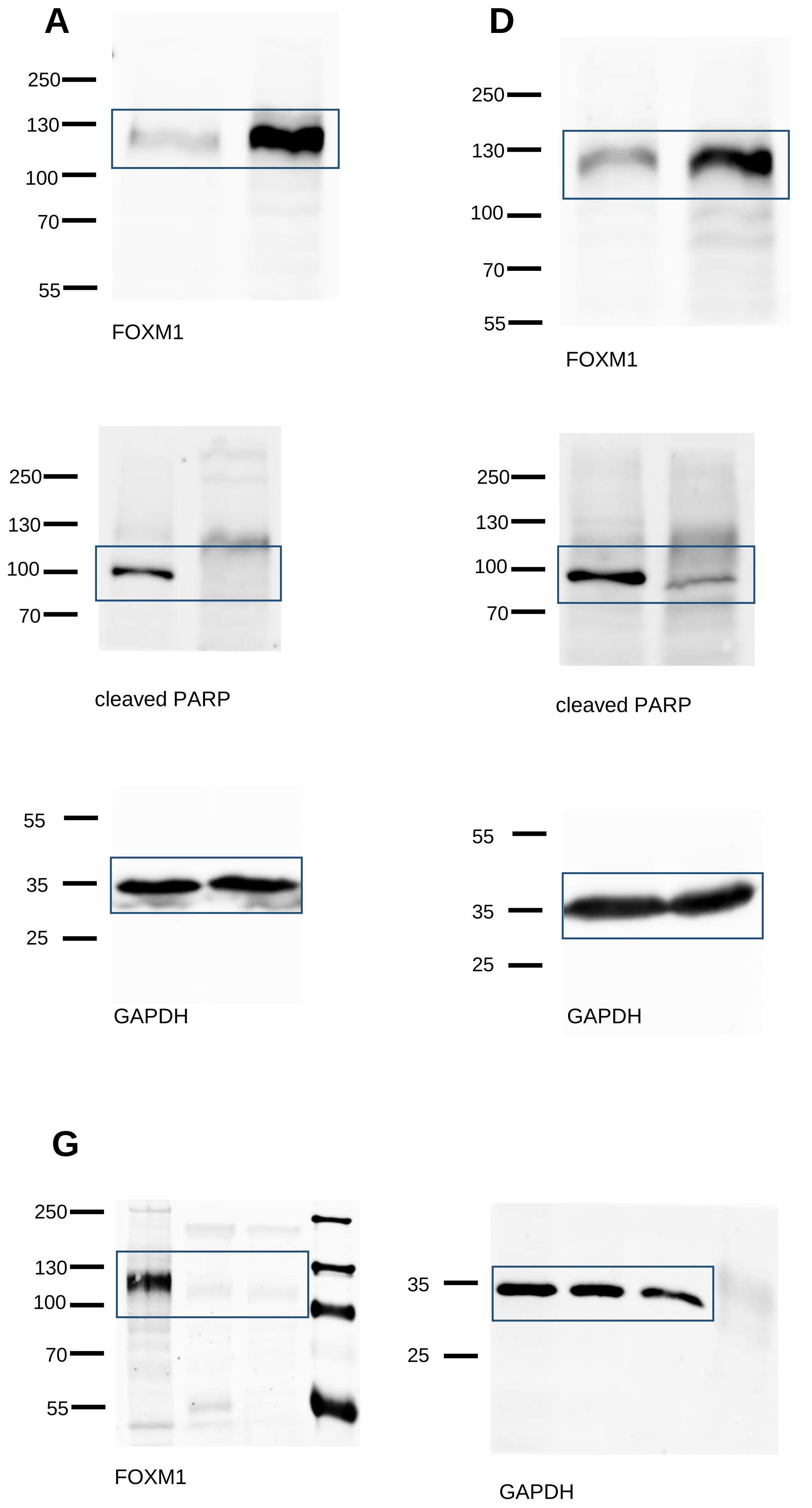

Supplementary Figure 6

E

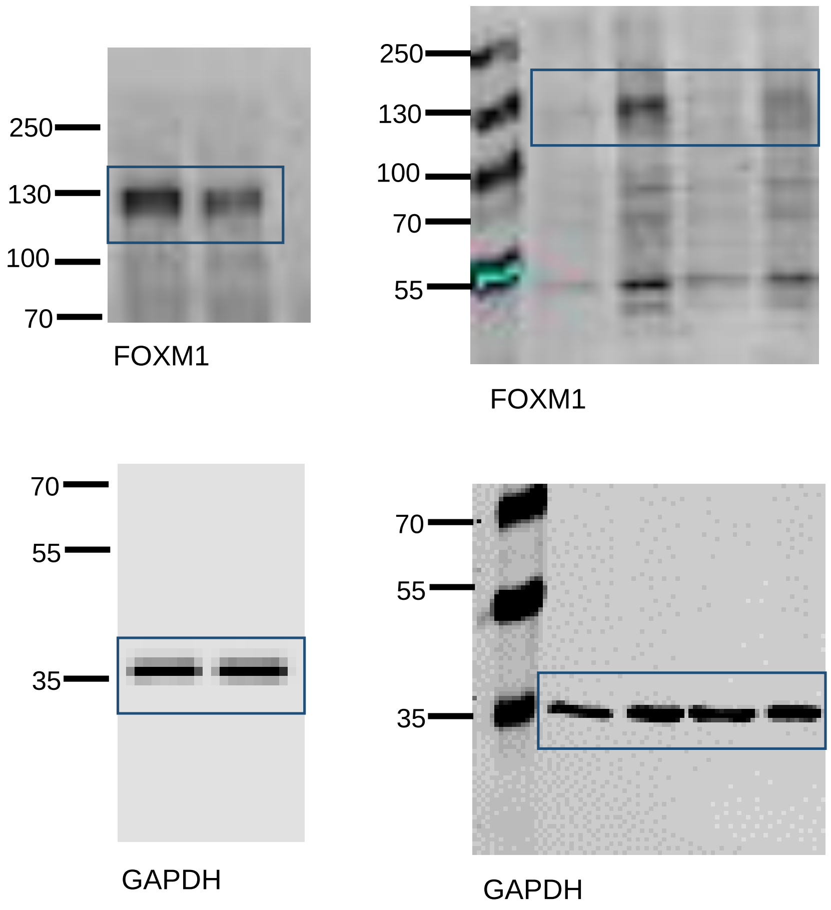

H

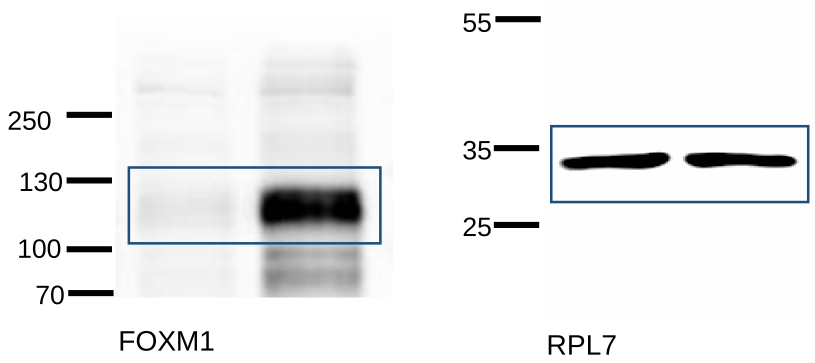

I

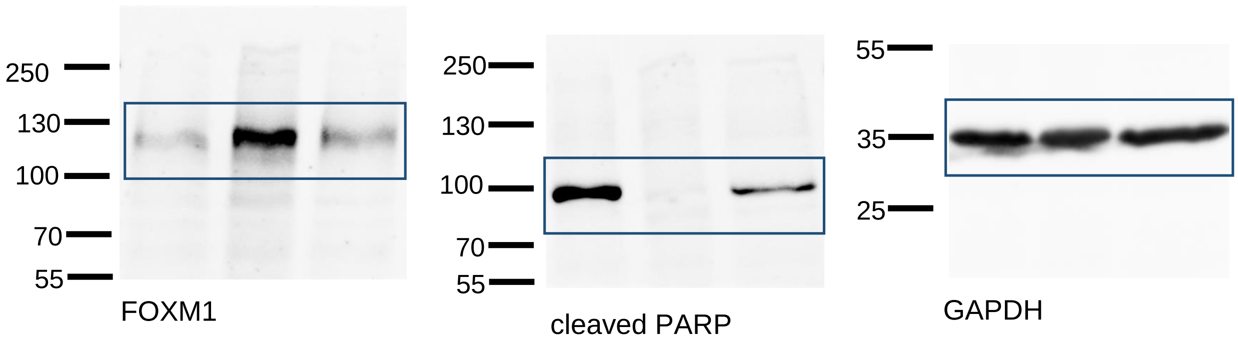

Supplement: Supplementary file 3 — Supplementary Material 3 [file 12964_2025_2644_MOESM3_ESM.pdf]
